# Using OpenStreetMap point-of-interest data to model urban change—A feasibility study

**DOI:** 10.1371/journal.pone.0212606

**Published:** 2019-02-25

**Authors:** Liming Zhang, Dieter Pfoser

**Affiliations:** Department of Geography and GeoInformation Science, George Mason University, Fairfax, VA, United States of America; IT University of Copenhagen, DENMARK

## Abstract

User-generated content is a valuable resource for capturing all aspects of our environment and lives, and dedicated Volunteered Geographic Information (VGI) efforts such as OpenStreetMap (OSM) have revolutionized spatial data collection. While OSM data is widely used, considerably little attention has been paid to the quality of its Point-of-interest (POI) component. This work studies the accuracy, coverage, and trend worthiness of POI data. We assess the accuracy and coverage using another VGI source that utilizes editorial control. OSM data is compared to Foursquare data by using a combination of label similarity and positional proximity. Using the example of coffee shop POIs in Manhattan we also assess the trend worthiness of OSM data. A series of spatio-temporal statistical models are tested to compare change in the number of coffee shops to home prices in certain areas. This work overall shows that, although not perfect, OSM POI data and specifically its temporal aspect (changeset) can be used to drive urban science research and to study urban change.

## 1 Introduction

The inexorable trend towards urbanization worldwide presents a pressing challenge to our understanding of the scale and speed of urban processes. *Scale* refers to the quantity of data involved given the changing spatial and temporal resolution of the data. Traditionally, urban theories examine a coarse level such as a whole city observed over decades. Nowadays we try to understand the urban processes at the building and the citizen level. This trend creates a considerable data science research challenge. Complementing scale, *speed* refers to the analysis of data in real-time, which should lead to faster and more responsive decision making. These aspects give rise to the emerging data science field called *Urban Analytics*. As part of Urban Analytics we use novel types of data to evaluate contemporary and future cities through methods including GIS, Remote Sensing, Big Data and Geodemographics [1]. In studying *urban change*, we try to assess and verify urban theories using data science methods. Urban change relates to the physical environment [2], including land use [3], infrastructure, business locations [4] and other assets of a city. Such an effort necessitates basic data collection, which until recently has been the responsibility of public authorities. This creates a bottleneck with respect to scale and speed, since such efforts rely on considerable man power and funding support. Updates to the data follow a 5+ years collection cycle. Studying urban change at the levels of granularity outlined above, requires data of a higher spatial and temporal resolution. Depending on existing collection infrastructure and regulations, such data might not be available and/or trustworthy [5].

Our work tries to assess the suitability of user-generated content in the form of OpenStreetMap POI data as a means to infer urban change. Specifically, we will explore two aspects of the data: (i) accuracy and coverage and (ii) trend worthiness. The *accuracy and coverage* of OSM POI data is not always evident and has been the focus of a sizeable research community over the years (cf. Section 2). In this work, we compare OSM POI data to Foursquare data. Foursquare data although a crowdsourced resource, exhibits some editorial control. Our comparison will use the name and location of POIs in both sources to reason about the respective coverage. Besides positional accuracy, a question we would like to answer is whether VGI is updated in a timely manner and can be used to capture trends and, in our specific context, urban change. To satisfy this so-called *trend worthiness* criterion, we assess whether VGI can match some commonly recognized trends or theories. Often urban analytics study the interaction of an environment to social phenomena. Our approach here is that by using statistical modeling, we consider VGI as trend worthy if the *modeling error is acceptable while maintaining its statistical behavior*. Such a test is useful in a social science context, since it would conditionally permit the use of VGI and, more generally, user-generated content in an otherwise data-poor environment. To this effect, we create statistical models based on population-based power law relationships in urban science [6]. This underlying theory suggests that population growth is one of the fundamental parameters behind change to an urban environment. For example, some basic power-law functions can relate population size to urban phenomena such as electricity usage, salaries, road network length, intellectual output, and even the citizens’ average speed of walking and heart rate. With evaluate a range of models in terms of their quality and how well they allow us to use OSM POI as an indicator of urban change.

The remainder of this work is organized as follows. Section 2 discusses related work such as Power Law Relationships and how they capture urban phenomena as well as quality aspects and fitness-for-use of user-generated content. Section 3 describes the data sources, related collection methods, and provides some data visualizations. Section 4 outlines our methodology to assess data quality. Section 5 presents the results of the quality assessment, including error distribution, the performance of statistical modeling, and interesting observations such as “inverse coffee shop effects”. Section 6 concludes and gives directions for future research.

## 2 Related work

Our discussion of related work focuses on two aspects, (i) user-generated content and its quality and fitness-for-use issues, and (ii) urban science theory as related to our work.

### 2.1 User-generated content and quality

Given the advent of volunteered geographic information (VGI) [7] and geospatial crowdsourcing [8], more and more relevant user-generated content becomes available. The most well-known geo-crowdsourcing effort is OpenStreetMap (OSM) (http://www.openstreetmap.org), colloquially referred to as the Wikipedia of Maps. OSM is a “free” vector dataset covering the entire planet. It has started out as a road network dataset, but by now includes general spatial feature information (transportation networks, buildings, land use data) and, important for this effort, point-of-interest data. A concern with VGI and user-generated content is data quality. Given the lack of quality control in data collection, the error in the data could be wild. OSM coverage and accuracy are for example examined in [9–12]. The work in [9] examines the accuracy and coverage of OSM by comparing it to British Ordnance Survey datasets. In this case, the error of OSM was found to be within 6*m* and the data had a 26% coverage. It should be noted that this study was conducted almost a decade ago. The authors of [11] assessed spatial coverage and ground-truth positional accuracy for five cities and towns in Ireland by comparing OSM data to Google and Bing maps. No method is proposed for POI data. A more systematic quality assessment is conducted in [12], which proposes metrics for geometric, attribute, semantic and temporal accuracy, as well as logical consistency, completeness, lineage, and usage. Most work however has focused on road networks and considerably little attention has been paid to POI data.

A recent OSM use cases survey [13] mentions an increasing number of data mining efforts in support of urban analytics. Urban form and function is but one area. [14] discusses different aspects of urban form and function and how they can be captured from user-generated content. Examples include map construction algorithms [15–17] and social media mining methods [18]. [5, 19] developed algorithms to better infer land use from OSM. Some other studies evaluated the overall potential for using OSM to assess land use and land cover [20, 21]. Another study in [22] presents an Urban-Rural Index derived from OSM to create an objective understanding of rural and urban classification. To quantify urban change, business directories and geocoding have been used in various efforts [23–26].

### 2.2 OSM POI quality assessment

Focusing on city scale, various authors have examined POI data quality. In [27], the authors propose a *spatial-semantic interaction* methodology to analyze the internal reference of different types of features of OSM POIs. The method developed so-called variograms and clusters of different feature types. The similarity of two POI features is defined as the spatiotemporal co-occurrence of different feature types of the same POI. A spatial process statistic is calculated to see if the processes are independent or not. Similar to our challenge, the authors mention that a weakness in their approach is the absence of a reference dataset. Recently, fitness-for-use of POIs [28] is defined at the levels of geo referencing, i.e., is the location accurate and allows for inferring an unambiguous reference to a real-world entity. Unfortunately, the authors do not propose a systematic metric for their measure. An overview paper [29] compares different aspects of the quality of OSM data, such as coverage, accuracy, and historical change using methods developed by different researchers. This work includes a reference dataset, the BD TOPO database produced by IGN, and it compares the data to a dataset derived from Flickr. The ambition of this quality assessment is limited to accuracy and coverage. To the best of our knowledge, our work is the first study that assesses the “fitness-for-use” of OSM POI data for studying urban phenomena and change. While we also consider accuracy and coverage, our most important contribution relates to the examination of *trend worthiness* of OSM POI data as a means to assess urban change. We consider a POI dataset trend worthy if the relative rate of change in the number of POIs accurately reflects a change in the real world, while conceding that the number of POIs at any given time deviates from the actual number of POIs existing in the real world, i.e., actual coffee shops vs. coffee shop POIs recorded in OSM.

### 2.3 Power law relationships

The fundamental dynamics of cities can be related to power law scaling relationships [6], i.e., urban phenomena scale with population size based on an universal power law function *P* = *αN*^*β*^, in which *P* is a specific kind of urban indicator, *N* is population size, *α* and *β* is scaling parameters. Based on the value of *β*, we can develop several growth models, which introduce different rates of growth for different cities. The authors here draw a comparison between the size of cities and the size of life forms and the energy demand each has to be sustainable. This work lead to countless results utilizing this theory, including the shape of cities [30], mobility patterns [31], and urban metabolism theory [32].

Other works have examined coffee shops and their deep connection to our social life. Coffee shops are social capital [33], in that a good coffee shop can provide a stronger sense of community for the residents of a neighborhood. One can consider places such as coffee shops, plazas, market places etc. as “the heart of a community’s social vitality and the grassroots of democracy” [34]. They represent a “Third Place” and are considered an essential part of society besides the workplace and home. With this social view of urban spaces, [35] provides a detailed review of how computing and analytics are augmenting people’s experiences of cities. The author argues that urban sensing and analytics will lead to considerable urbanization improvements. Some quantitative studies have empirically evaluated these visions. The authors of [36] investigate the effect of coffee shops on crime. The authors argue that coffee shops provide “an on-the-ground and visible manifestation of a particular form of gentrification… and lifestyle.”

With this theoretical background, we are confident in the use of power law relationships when using the change of POI data to study urban phenomena. *We would like to point out that this work should not be considered the one and only approach to studying urban change, but rather proposes a method that leverages OSM POI data in this context*.

## 3 Data

Various datasets are used to provide for a sound quality assessment of OSM POI data. We assess (i) the accuracy and coverage of OSM as a POI data source by using/comparing it to respective Foursquare data, and we assess (ii) its trend worthiness by building statistical models that relate the change in coffee shops to a change in home prices. The following sections discuss each data source as well as the pre-processing steps needed to make the data actionable.

### 3.1 OSM coffee shop data

**Fig 1:**
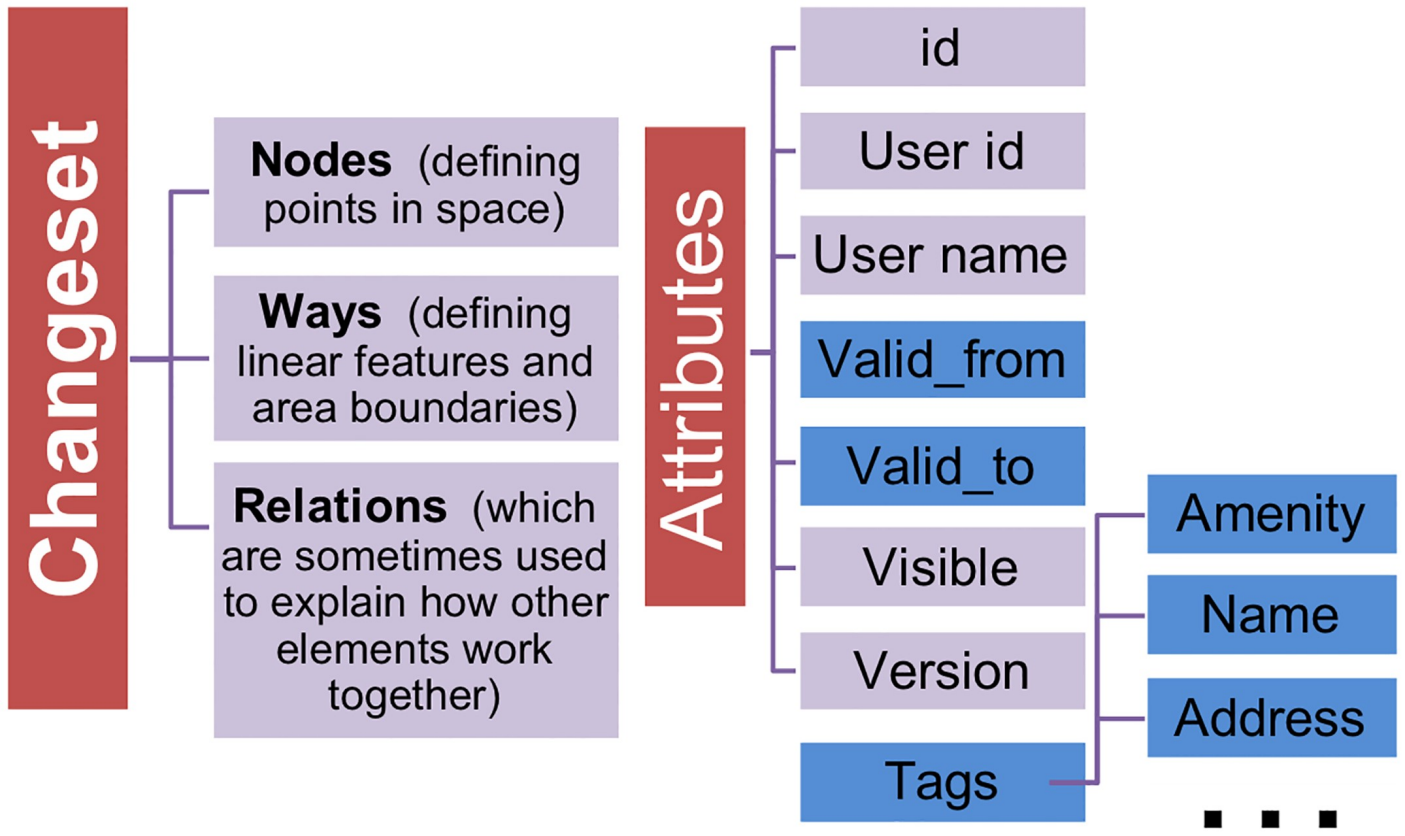
OSM‘s changeset data object model.

OSM allows anybody to freely edit a global map dataset. To keep track of the edits, OSM uses an independent data object called “changeset” to record changes from editing operations and users. Changesets record all changes such as tags, coordinates, and comments. Such a database then includes for all OSM objects (nodes, ways, relations) respective metadata information as shown in Fig 1).

**Fig 2:**
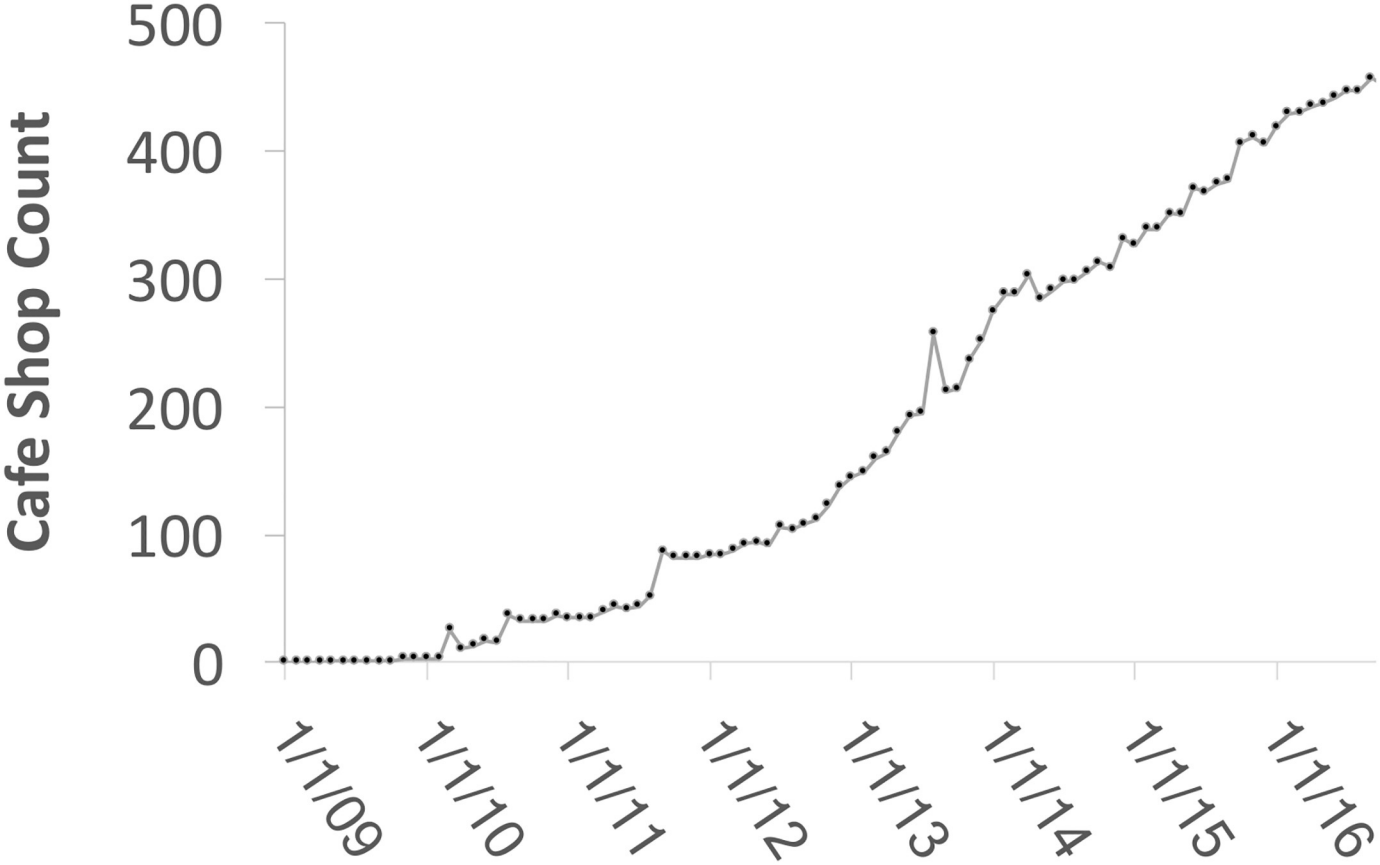
Coffee shop count in Manhattan from OSM—Monthly 1/1/2009–11/31/2016.

For our work, we are interested in changeset data that reflects actual real-world change, i.e., the addition or deletion of a coffee shop node in close temporal proximity to the actual opening or closing of the coffee shop. Since we do not have ground truth data, i.e., municipal records, we use quantitative information such as the monthly coffee shop count plot in Fig 2, which shows the overall change in OSM data.

Assuming OSM has matured as a dataset, the plot shows a slow growth in numbers after OSM’s inception, as not too many people were aware of it. Following 2010, the rate of growth in edits increases and after 2015 the growth slows again, suggesting that the POIs recorded in OSM start reflecting real world changes, i.e., coffee shops that opened were recorded in a timely manner. Based on these trends, we select coffee shop changesets for the period of 11/2014 to 11/2016 for our experimentation and study.

**Fig 3:**
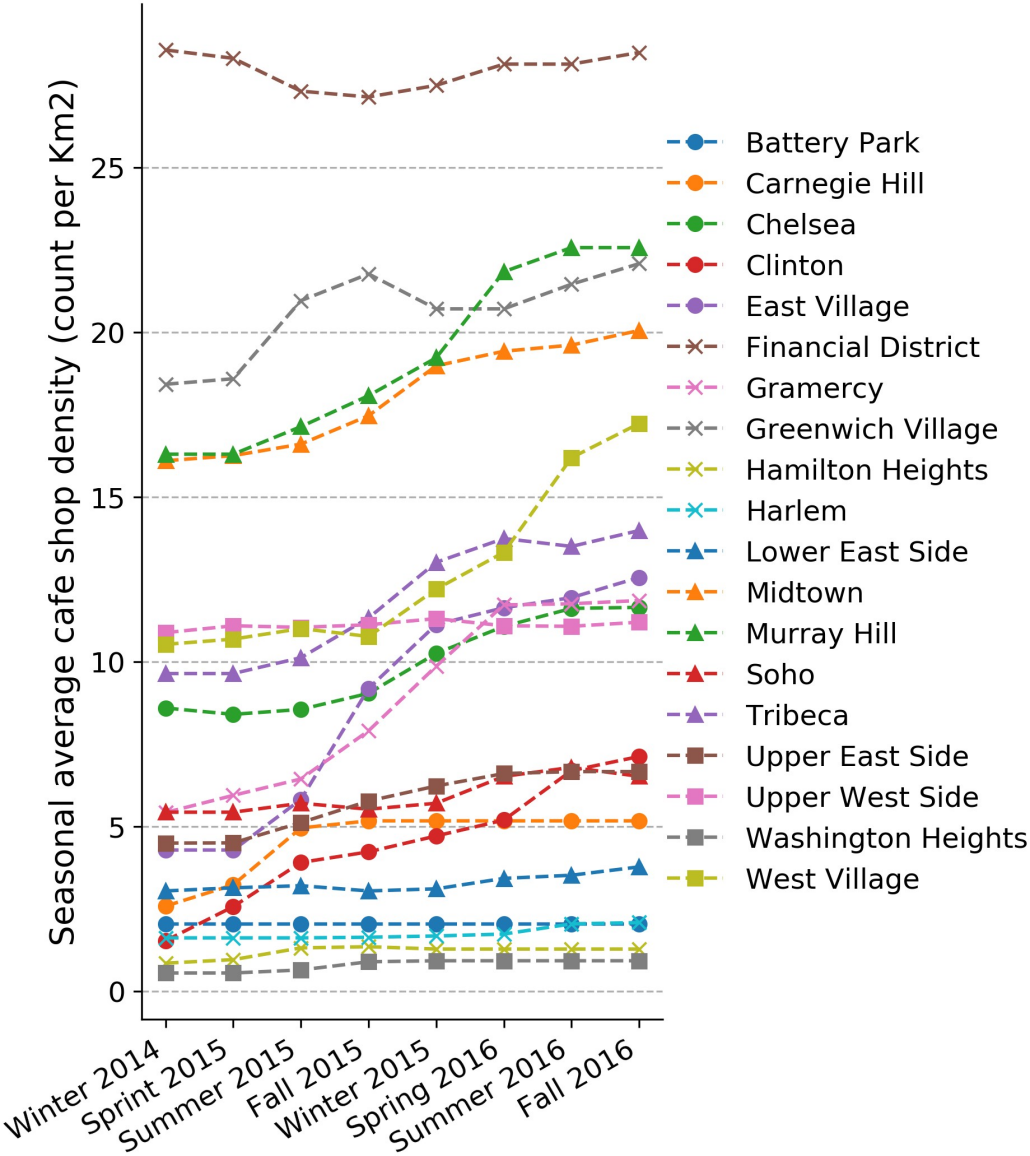
Coffee shop density [*km*^2^]—Seasonal average 03/2015–11/2016.

**Fig 4:**
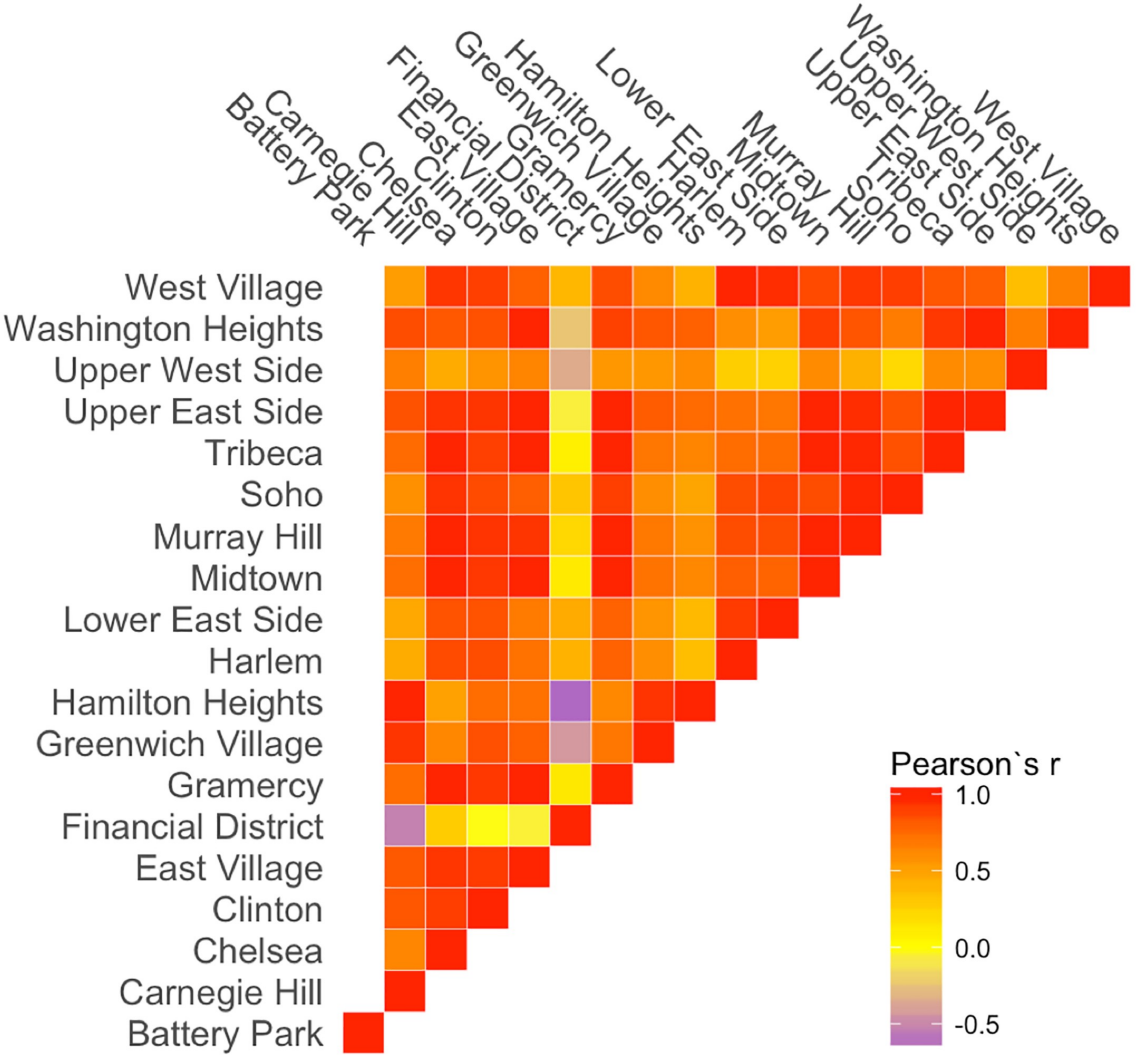
Pairwise Pearson correlation of neighborhoods’ coffee shop densities.

In a first step, we compute a seasonal average. Here, November, December, and January are defined as Winter, and so forth. Although not strictly correct, this definition has been used in [37] reporting on real estate pricing trends. We can define the seasonal coffee shop density as the number of coffee shops in a neighborhood divided by the area of the neighborhood (*km*^2^). We will use the term “Coffee Shop Density” in the remainder of this work. The changes to coffee shop density for each season are shown in Fig 3. We see that different neighborhoods seem to have different trends. While in most cases the coffee shop density is increasing, some neighborhoods, such as the Financial District and Soho, seem to suffer through periods of decreasing numbers. To better show different patterns between pairs of neighborhoods, a pairwise Pearson correlation (*r* score) [38] is shown in Fig 4. About half of the neighborhood pairs are strongly correlated (Pearson’s *r* > 0.5). Only the Financial District is negatively correlated with most of the other neighborhoods. This mix of trends could also be an indication for the reliability of OSM data, i.e., the closing of shops is actually reflected in the crowdsourced OSM dataset.

### 3.2 Foursquare coffee shop data

We use Foursquare data [39] as a means to verify the coverage of OSM POI data. Foursquare, a location-based social-media app, utilizes POIs as the location where the user “socializes” with friends. Users are encouraged to review and visit different locations as often as possible to claim them. Since POIs are a core data aspect of this app, it also exerts editorial control, i.e., POIs are actively curated, As such, we can consider this dataset a good reference dataset when it comes to evaluating crowd-sourced OSM data.

**Fig 5:**
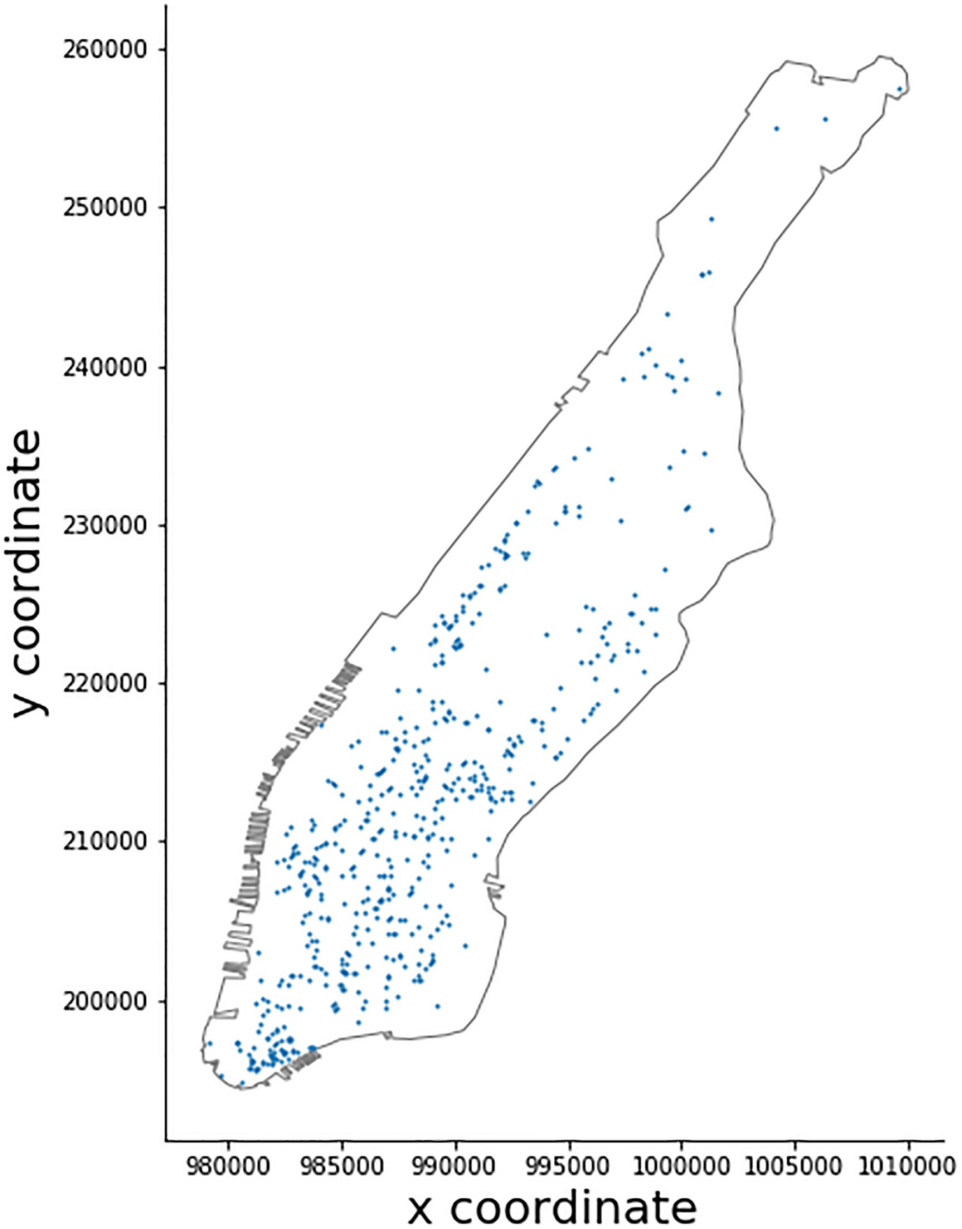
OSM coffee shop locations.

**Fig 6:**
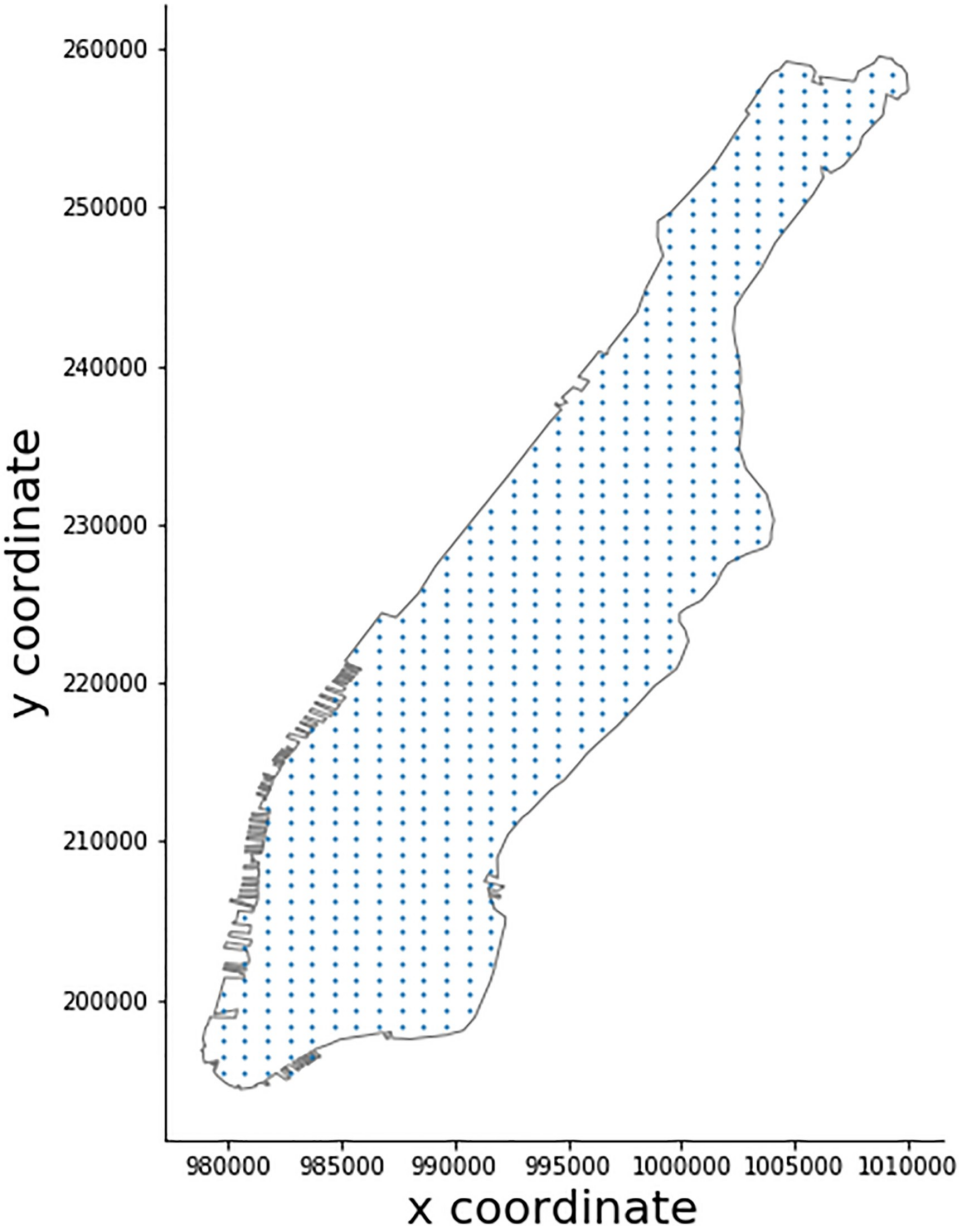
API search grid points.

Unfortunately, Foursquare does not provide access to their entire database in a fashion similar to OSM. An API with limited service rates allows one to interact with the service and in our case to retrieve POI information. To account for some API limitations, we use two types of queries as detailed in Tables A and Table B in S1 Appendix. Type I uses the collected OSM data to retrieve all Foursquare POIs that have a matching label within a 50*m* radius. Type II uses a regular spatial grid (200*m* spacing) to retrieve all coffee shop POIs in relation to the centroid of each cell. This strategy ensures that less than 50 POIs are retrieved per request (Foursquare limit), while it also covers POIs that are not in the immediate area of our OSM POIs. The mapping of categories of OSM data and Foursquare data is shown in Table C in S1 Appendix. The resulting Foursquare POI dataset is obtained by fusing these two datasets. The location of all OSM POIs for query Type I and grid center points for Type II are shown in Figs 5 and 6.

In total, 851 Foursquare POIs were retrieved on June 30, 2018. Section 4 will show how Foursquare and OSM POIs match up. An interesting observation is that Foursquare has a very different definition of POI categories when compared to OSM. For example, while Foursquare has a “donut” category, OSM considers it “cafe” (Table C in S1 Appendix).

Ideally our ground-truth data should have a historical dimension. Unfortunately, neither Foursquare nor any other data sources besides OSM captures this aspect. As such, we are only able to compare the accuracy of the current POIs between sources.

### 3.3 Home prices

**Fig 7:**
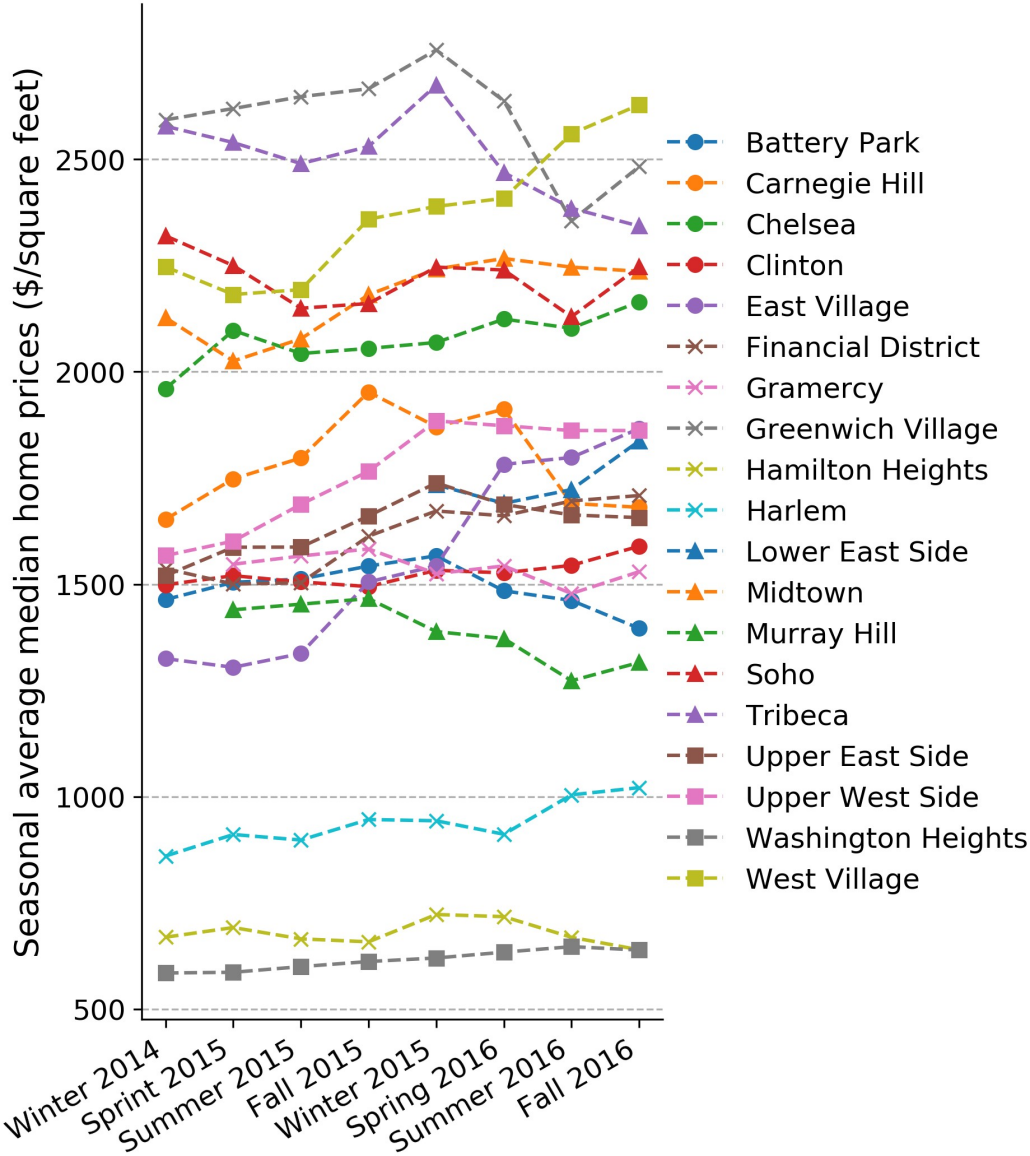
Unit home price (UHP) ($/*ft*^2^) changes—Seasonal average 12/2014–11/2016.

**Fig 8:**
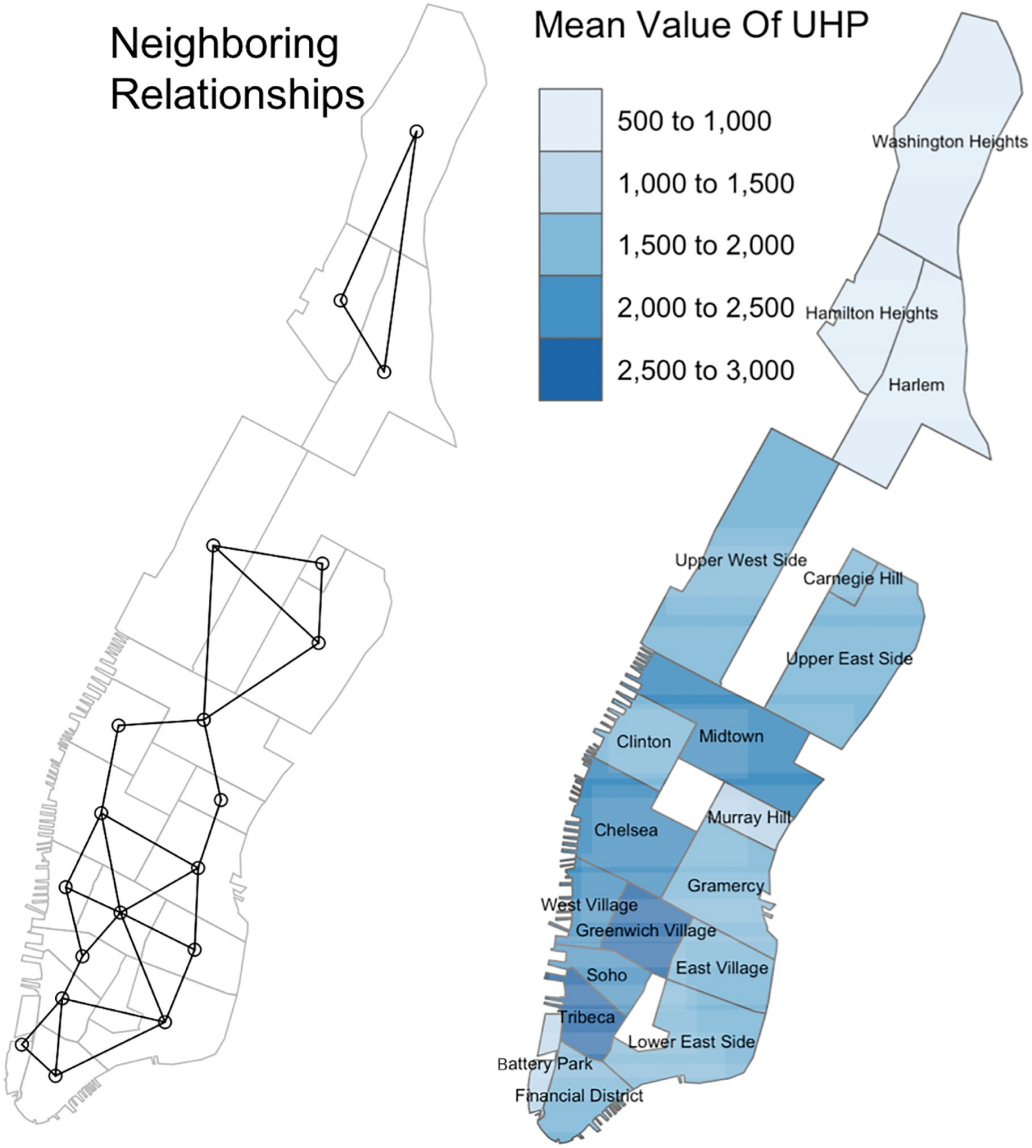
Relationships and mean values: (left) adjacency relationship map of neighborhoods (dots represent neighborhoods, edges indicate adjacent neighborhoods), (right) mean values of average unit house price (UHP).

To model the relationship between coffee shop densities and home prices, we obtain a dataset from Zillow, a real estate listing Web site. Zillow provides a data analysis product called “Home Value Index” (https://www.zillow.com/research/zhvi-methodology-6032/), which captures home prices at different spatial granularities ranging from city to neighborhood levels. This data is based on listing prices posted on the web site, and as such, can also be considered crowdsourced. For Manhattan, different datasets are provided at the neighborhood level. We selected the “Median List Price Per Sq Foot” from the Home Value Index. This data is more resilient with respect to outliers and removes a house size bias. We calculate the seasonal average of “Median List Price Per Sq Foot”, to which we refer to as home prices in the remainder of the paper. The fluctuations of this measure are shown in Fig 7. Seeing this data spatially, we observe that adjacent neighborhoods tend to have similar home prices. Fig 8 visualizes this relationship. Neighborhoods for which no data is available from Zillow or if they have no real estate market (Central Park) are left blank (white).

## 4 Methodology

**Fig 9:**
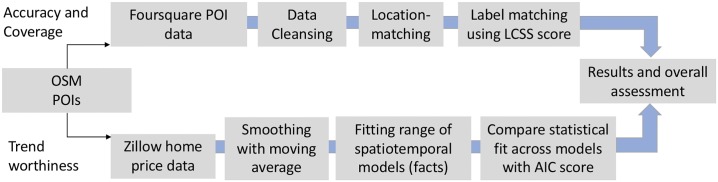
Quality assessment workflow overview.

Our main objective is to assess the quality of user generated content and argue for its use in urban science research. The following sections provide a detailed discussion of the overall methodology that is employed. Fig 9 provides and overview.

### 4.1 POI accuracy and coverage assessment

To assess the accuracy and coverage of OSM POI data, we compare it to a reference dataset. However, since there are no authoritative datasets available, we chose Foursquare POI data, which is crowdsourcing data with some editorial control. Unfortunately, no historical versions of the data are available. Our dataset was retrieved on June 30, 2018. Comparing two POI sources that cover the same geographic area (Manhattan) and context (coffee shops) is not trivial, given a potential label mismatch (“Ben’s Cafe and Eatery” vs. “Ben’s”) and location uncertainty (a daunting problem for any user-generated content). In the following, we try to match POIs from both data sets using string similarity measures and location proximity.

#### 4.1.1 Label similarity

To compare POI labels between data sources, we selected the Longest Common Sub-sequence (LCS) method and the Levenshtein distance (cf. [40]). The Levenshtein distance has been used in similar contexts, e.g., [29]. LCS gets its advantage over Levenshtein distance since it measures the difference of two strings that are compared to a common substring, while the Levenshtein distance uses one string as its reference. Given two crowdsourced data sources, it is unlikely that two labels for the same POI match up exactly. Both methods can provide an approximate match and can account for labels of varying length and spelling differences. Both measures are distance measures, which need to be converted to a similarity score. Similarity scores can be mapped to the interval [0, 1]. To formally define the similarity of two labels, the two *Label Similarity Scores* are defined as follows.

$$\begin{array}{c} S_{LCS}=\frac{L_{LCS}*2}{L_{FSQ}+L_{OSM}} \end{array}$$ (1)

$$\begin{array}{c} S_{LD}=\frac{L_{OSM}-L_{LD}}{L_{OSM}} \end{array}$$ (2)

Here, *L*_*LCS*_ is the length of longest sub-sequence and *L*_*LD*_ is the number of changes needed to transform one label to another (reference). *L*_*FSQ*_ and *L*_*OSM*_ capture the lengths of the Foursquare and OSM labels, respectively. Identical labels generate a score of 1 in both cases. Both functions are monotonically increasing as the length of a common sub-sequence increases (LCS), or the difference between strings decreases (LD), which makes both a valid and good similarity measure.

When calculating similarity, we have to consider some particularities of the data. In both datasets, common terms are “coffee”, “cafe”, and “cafeteria”, all referring to the same concept. Given the same POI, it might be labeled as “Starbucks” in one dataset and “Starbucks Coffee” in the other. In this case, its similarity scores would only be $S_{LCS}=\frac{9*2}{9+25}=0.529,S_{LD}=\frac{25-9}{25}=0.64$. As such we removed all commonly used terms from the labels to provide for a fair assessment (stop words). In addition, we also removed all white spaces and punctuation. The experiments use a rather strict similarity threshold of 0.9 for both methods (cf. Section 5).

#### 4.1.2 Location similarity

POIs are typically captured as point locations and the expectation is that the recorded coordinates do not match up exactly across data sources. To examine whether two coordinates capture the same POI (and without considering the label) one can use a buffer region to see whether one location is close to the other. The choice of a proper threshold is critical, as various coffee shops with the same name (chain) might be close by. Using a projected coordinate system, Euclidean distance can be used. Fig 9 gives an overview of the overall processing pipeline.

### 4.2 Trend worthiness of OSM POI data

While the expectations are that OSM data is timely and has considerable coverage, it might not always perfectly match the real-world situation, i.e., accuracy and coverage might not always reflect reality. As such we want to assess whether such data captures overall urban trends and change using a statistical modeling approach.

We first introduce the concept of scaling relationships to model urban phenomena based on population size. This allows us then to relate change in coffee shop numbers and home prices to population and to eventually model the direct relationship between them. We use a range of spatial and temporal analysis methods and adjustments to try and improve the overall model fit. Section 5 will finally tell us the adjustments that work best and consequently the model that has the best fit.

#### 4.2.1 Scaling relationship based on population

The fundamental driving force behind urban change is human activity and population size. In [6], it is shown that larger metropolitan areas produce comparatively more wealth, innovation and activity following a power law function based on population size.

This power law scaling relationship is shown in Eq 3. The variance of each urban indicator is the exponential scaling factor *β*, which can be used to derive three types of urban phenomena. With (i) *β* ≈ 1 (linear growth) we can describe individual human needs, like jobs and water consumption, (ii) *β* < 1 (sublinear growth) characterizes infrastructure, and (iii) *β* > 1 (superlinear growth) signifies quantities related to social currencies, like innovation. The coefficient estimation is done using linear regression after a log transformation of the scaling relationship model (Eq 4) [6]. In the following equations, *N*_*t*_ is the population at a certain time, *Y*_0_ is the initial state of an indicator, *Y*_*t*_ is the current state of an indicator, and *ϵ*_*t*_ is the error.

$$\begin{array}{cc} & Y_{t}=Y_{0}N_{t}^{\beta } \end{array}$$ (3)

$$\begin{array}{cc} & \text{log}\left(Y_{t}\right)=\text{log}\left(Y_{0}\right)+\beta \;\text{log}\left(N_{t}\right)+\epsilon_{t} \end{array}$$ (4)

Coming back to our problem of coffee shops and home prices, in [36] the authors found that coffee shops are a good indicator for gentrification. Gentrification is a common and controversial topic in politics and urban planning and refers to improving deteriorated urban neighborhoods by an influx of a wealthier demographic. Intuitively, the establishment of a coffee shop relies on certain population numbers (customers) to support it. To open a coffee shop, a much smaller investment is needed than, for example, a super market. As such, *the number of coffee shops is sensitive to relatively small changes in population numbers (volatility)*. It is easier to close a coffee shop than a supermarket when customers stay away. Another argument here is that coffee is a cheap commodity that is consumed frequently. Hence, it is not as susceptible to personal spending cuts as more expensive products such as entire meals, clothing or jewelry.

**Power low relationship between coffee shops and home prices**. Coffee shop numbers correlate with population numbers over time and place and can be treated as an indicator of human activity. Thus, they should follow a power law function of population. On the other hand, the real estate market is an economic phenomenon also related to human activity. With Eqs 3 and 4, we have two relations between coffee shops *c* and population, and between home prices *p* and population: log(*c*_*t*_) = *log*(*c*_0_) + *β*_*c*_ log(*N*_*t*_) and log(*p*_*t*_) = *log*(*p*_0_) + *β*_*p*_ log(*N*_*t*_). In combining them, we infer a function between coffee shops and home prices. Eq 5 also represents a power low scaling relationship and can be fitted using regression techniques. As a side benefit, since both datasets are user generated, it would also establish the usefulness of such data for the investigation of urban phenomena.

$$\begin{array}{c} \begin{array}{cc} \text{log}(P_{t}) & =\tau +\beta \;\text{log}(C_{t})\\ \tau & =log\left(P_{0}\right)-\;\beta_{P}/\beta_{C}\;log\left(C_{0}\right)\\ \beta & =\beta_{P}/\beta_{C}\; \end{array} \end{array}$$ (5)

In Eq 5, log(*P*_*t*_) is the log transformed home price and log(*C*_*t*_) is the log transformed coffee shop density. In the remainder of this paper, these log transforms are still referred to as “coffee shop density” and “home price”.

**Fig 10:**
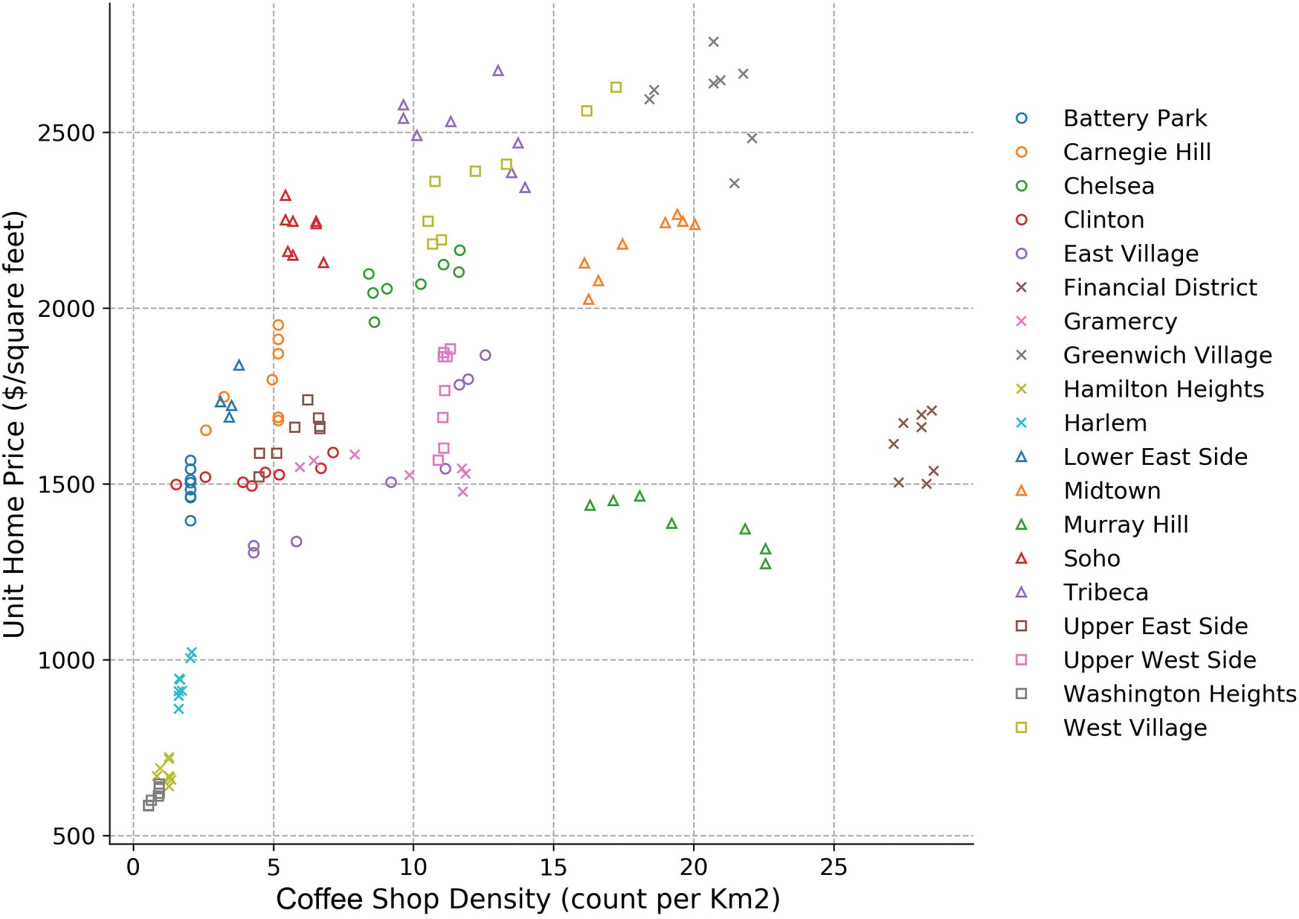
Log(coffee shop density) vs. log(unit home price) grouped by neighborhoods.

**Fig 11:**
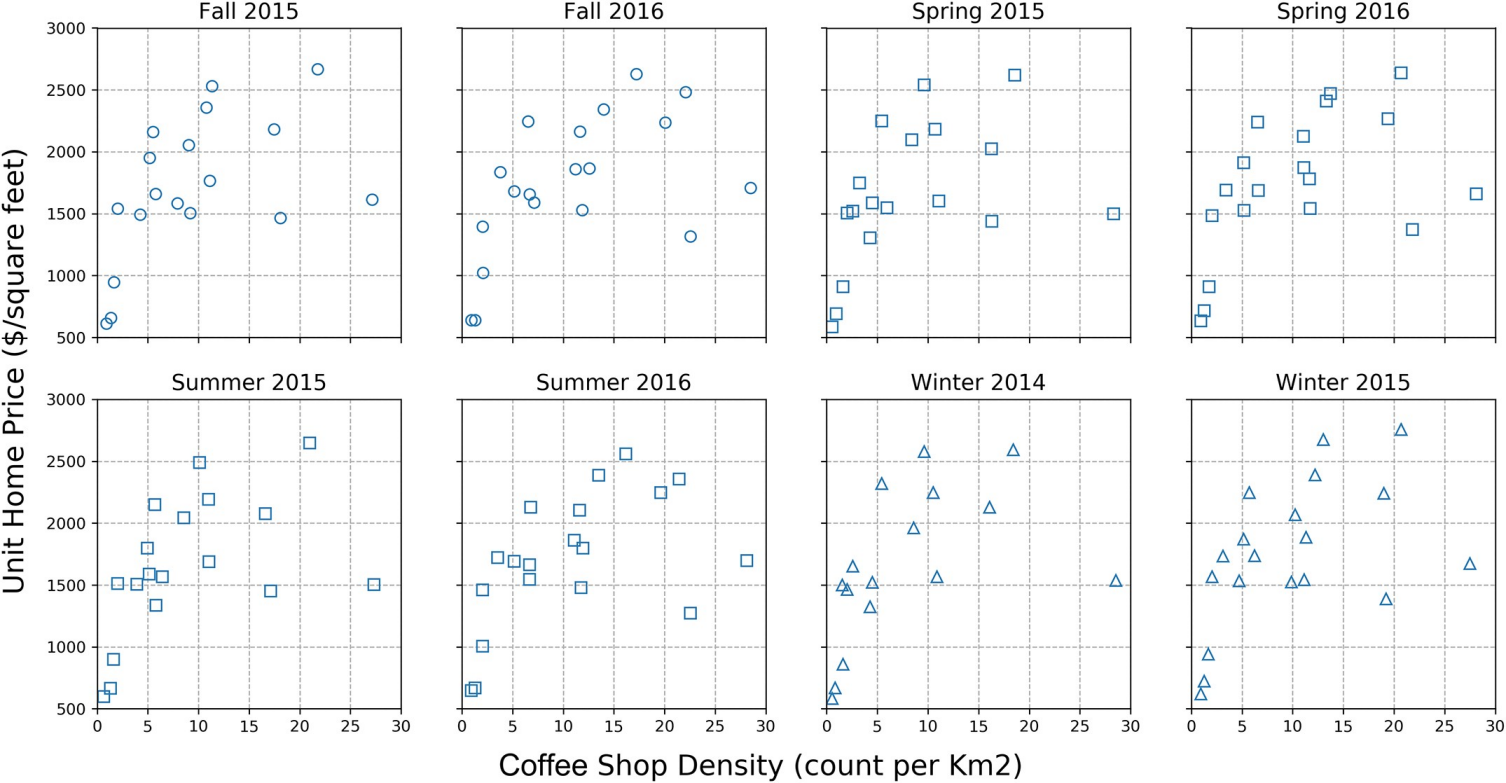
Log(coffee shop density) vs. log(unit home price) grouped by seasons.

Fig 10 shows that different neighborhoods over time (shown using different colors and symbols) exhibit different patterns. The home prices of each neighborhood do not always increase as the coffee shop density increases. Fig 11 shows seasonal patterns across all neighborhoods and they seem to be more consistent. Different sub-figures represent different seasons in different years. Home prices seem to increase with coffee shop density for each season in general. In the lower-left corner, both, coffee shop density and home prices are low. In the upper-right corner, home price and coffee shop density have diverging trends.

These different patterns in both figures indicate that there might be other spatiotemporal effects at work beyond the basic scaling relationship model. We will use this model as our basic model and introduce a series of adjustments that utilize some commonly recognized temporal and spatial patterns in urban science.

#### 4.2.2 Temporal trend and lag

**Global trend**. Every city’s real estate market is affected by global market forces and the economy. The economic cycle is a commonly recognized trend [41] and refers to market fluctuations over certain periods, e.g., ten years. While there are many modeling techniques to estimate economic cycles, our data does only cover a two-year period of a typical ten-year cycle and as such it could cover any portion of the cycle (peak, bottom, etc.). We use the following general polynomial model for this trend: *τ* + *α*_1_*t* + *α*_2_*t*^2^. Here *t* is an index capturing sequential seasons ([1, 8] of our two year period), *τ* is the intercept term, *α*_1_ and *α*_2_ are coefficients. This function can generalize the situation mentioned before. If *α*_2_ is zero (or not statistically significant) this model is reduced to a linear trend model. If *α*_1_ and *α*_2_ are both zero (or not statistically significant), it becomes a stationary process.

Creating one model that captures all neighborhoods, we need to address the differences in magnitudes of prices existing in those neighborhoods. We observed that during a recession, the absolute devaluation of properties of higher value would be much more than the absolute devaluation of properties with lower value. The reverse can be said during boom periods. We use the mean value of the unit home price for the entire two year period $\bar{P_{n}}$, with *n* representing different neighborhoods to normalize home prices. The scaled polynomial model becomes $\tau +\alpha_{1}\bar{P_{n}}t+\alpha_{2}\bar{P_{n}}t^{2}$. The modified scaling relationship model is then as follows.

$$\begin{array}{c} \text{log}\left(P_{t}\right)=\tau +\beta \;\text{log}\left(C_{t}\right)+\alpha_{1}\bar{P_{n}}t+\alpha_{2}\bar{P_{n}}t^{2}+\epsilon_{t} \end{array}$$ (6)

**Seasonality**. Many articles investigating the real estate market discuss the “seasonality” of home prices (cf. [42–44]). Following a typical approach, seasonality is modeled using dummy variables ([45, 46]). Our model can be restated as follows.

$$\begin{array}{c} \text{log}\left(P_{t}\right)=\beta \;\text{log}\left(C_{t}\right)+\alpha_{1}\bar{P_{n}}t+\alpha_{2}\bar{P_{n}}t^{2}+\sum_{i}^{4}I_{\left(i\right)}w_{i}+\epsilon_{t} \end{array}$$ (7)

Here, *I*_(*i*)_ and *w*_*i*_ are seasonal dummy variables and their coefficients, respectively. With seasonal dummy variables present, the intercept term *τ* of the scaling relationship model is dropped from the linear model, since a collinearity issue would exist between *τ* and seasonality. Both seasonality and *τ* are implicitly estimated by *w*_*i*_. A larger *w*_*i*_ means a larger seasonality factor, and vice versa.

**Temporal lag**. The relationship between coffee shops and home prices could be that an increase of coffee shops either (i) leads to higher home prices, (ii) coincides, or (iii) follows home prices. As such, we can add a lag variable for coffee shop density to our model.

$$\begin{array}{c} \begin{array}{c} \text{log}(P_{t})=\beta \;\text{log}\left(C_{t}\right)+\alpha_{1}\bar{P_{n}}t+\alpha_{2}\bar{P_{n}}t^{2}+\sum_{i}^{4}I_{\left(i\right)}w_{i}+\sum_{j}^{3}\eta_{j}\;\text{log}\left(C_{t-j}\right)+\epsilon_{t} \end{array} \end{array}$$ (8)

In this new model, log(*C*_*t*−*j*_) is the last *j* season’s difference to the current season’s coffee shop density and *η*_*j*_ is its coefficient.

#### 4.2.3 Spatial trend and autoregression

**Spatial trend**. Many physical or social phenomena, such as the earth’s gravity, snow thickness, and population are correlated with their location. Using this basic spatial analysis technique, the spatial trend in observational data is described by means of a two-dimensional polynomial equation [47]. Despite some claims that it does not perform well for the case of real estate [48, 49], we still want to investigate its performance given that it is a basic geospatial method.

For Manhattan, (cf. Fig 8 right) it shows that home prices are higher in the south of the city than in the north. Two-dimensional coordinates themselves do not influence home prices. However, since many spatial phenomena in a city follow a certain spatial configuration, e.g., subway lines, tourism, etc., home prices might be affected by them and as such are implicitly correlated with location.

In our case of incomplete knowledge and variables, space might be a proxy for the missing information and spatial trend analysis might still be valuable for our model. Since coffee shop density could follow a spatial trend, a collinearity issue might exist between location and coffee shop density. The updated model using a polynomial for the *x* and *y* coordinates is as follows.

$$\begin{array}{c} \begin{array}{cc} \text{log}(P_{t}) & =\beta \;\text{log}\left(C_{t}\right)+\alpha_{1}\bar{P_{n}}t+\alpha_{2}\bar{P_{n}}t^{2}+\sum_{i}^{4}I_{\left(i\right)}w_{i}\\ & +\theta_{1}x+\theta_{2}y+\theta_{3}x^{2}+\theta_{4}y^{2}+\theta_{5}xy+\sum_{j}^{3}\eta_{j}\;\text{log}\left(C_{t-j}\right)+\epsilon_{t} \end{array} \end{array}$$ (9)

**Spatial autoregression model**. In this model we consider spatial autocorrelation, which is the interdependence between different locations. It is widely used in economy, sociology and biology when one needs to analyze the correlation of neighboring phenomena. It helps in establishing that neighborhoods sometimes exhibit similar characteristics at a certain spatial scale [50]. Recently, the authors of [51] argue that latent factors could be universal behind all of those autoregressive models. In a similar argument, spatial autocorrelation is linked to missing values estimation and interpolation [52]. For example, in the case of Manhattan there are some externalities, such as transportation hubs, parks, and venues that affect more than one neighborhood. From the perspective of latent factors, autoregression models estimate additional hidden parameters that can increase the goodness-of-fit of our model. This is especially the case if its residuals do not show the expected random distribution and they are spatially clustered creating spatial interdependence. There are two basic spatial autoregression models. Eq 10 is spatial lag based and includes lagged dependent variables. Eq 11 is spatial error based and includes lagged error terms (cf. [50]). In both equations, *Y*_*t*_ is the vector of observed home prices for each neighborhood at time *t* with dimension *n* × 1. *W* is a weighted matrix with dimension *n* × *n*. *X*_*t*_ is a matrix of regressors with dimension *n* × *m*, with *m* being the number of regressors in this model, including coffee shop density and the variables discussed in the adjustment models. *β* is the coefficients’ vector with dimension *m* × 1. *ϵ*_*t*_ is the error term. *ρ* is the coefficient of the spatial lag term. λ is the coefficient of the spatial error term.

$$\begin{array}{c} Y_{t}=\rho WY_{t}+\beta X_{t}+\epsilon_{t} \end{array}$$ (10)

$$\begin{array}{c} \begin{array}{c} Y_{t}=\beta X_{t}+\epsilon_{t}\\ \epsilon_{t}=\lambda W\epsilon_{t}+\mu_{t} \end{array} \end{array}$$ (11)

**Modeling approach**. In our study, we build models starting with a basic model up to involved models that include additional regressors. We do so in order to observe whether subsequent adjustments add power to the model. The simplest model uses a mean value of coffee shop density and home prices across all seasons. One model is built for each season. We use this simple approach to show the applicability of a scaling relationship model.

Next, a comprehensive model with data for all neighborhoods and seasons is introduced. It includes coffee shop density as an independent variable. Based on this model, different independent variables and spatial autoregression methods are added or deleted based on their respective p-value and modeling power.

To assess the performance of our various models, we choose the Akaike Information Criterion (AIC) [53] and the Bayesian Information Criterion (BIC) [54] instead of the typical R-squared metric since AIC and BIC are more resilient to overfitting [55]. In our modeling approach, there are two sources of complexity. The first is an increasing number of different independent variables. The second source comes from the fact that polynomial models have the potential risk of being overly complex for our modeling case. Both AIC and BIC are information-based criteria that assess model fit as metrics for selecting a finite set of models. They both maximize likelihood and penalize an increasing number of parameters and complexity. As such, both are resilient to overfitting and they are widely used for model comparisons in modern statistics. In general, the criteria for model comparison is that lower AIC or BIC values indicate a better model fit. Normally, BIC will give a higher score as it penalizes model complexity more than AIC. One strategy to add or eliminate a variable is that if a variable (i) is not considered statistically significant (p-value), or (ii) a model using it has a similar or worse AIC or BIC value than a simpler model, then this variable would not be included in the next (improved) model. Another strategy is that if coefficients of coffee shop density change significantly when new variables are added, the model is considered a bad one, since they eliminate the contribution of coffee shop density in our model. This reasoning relates to the discussion of spatial trends and spatial auto-regression and that these models might implicitly (location) estimate coffee shop density.

## 5 Experimental results

The ambition of this section is simple. We want to evaluate the POI Accuracy & Coverage and trend worthiness methods that we defined in Section 4.

### 5.1 Accuracy and coverage

**Fig 12:**
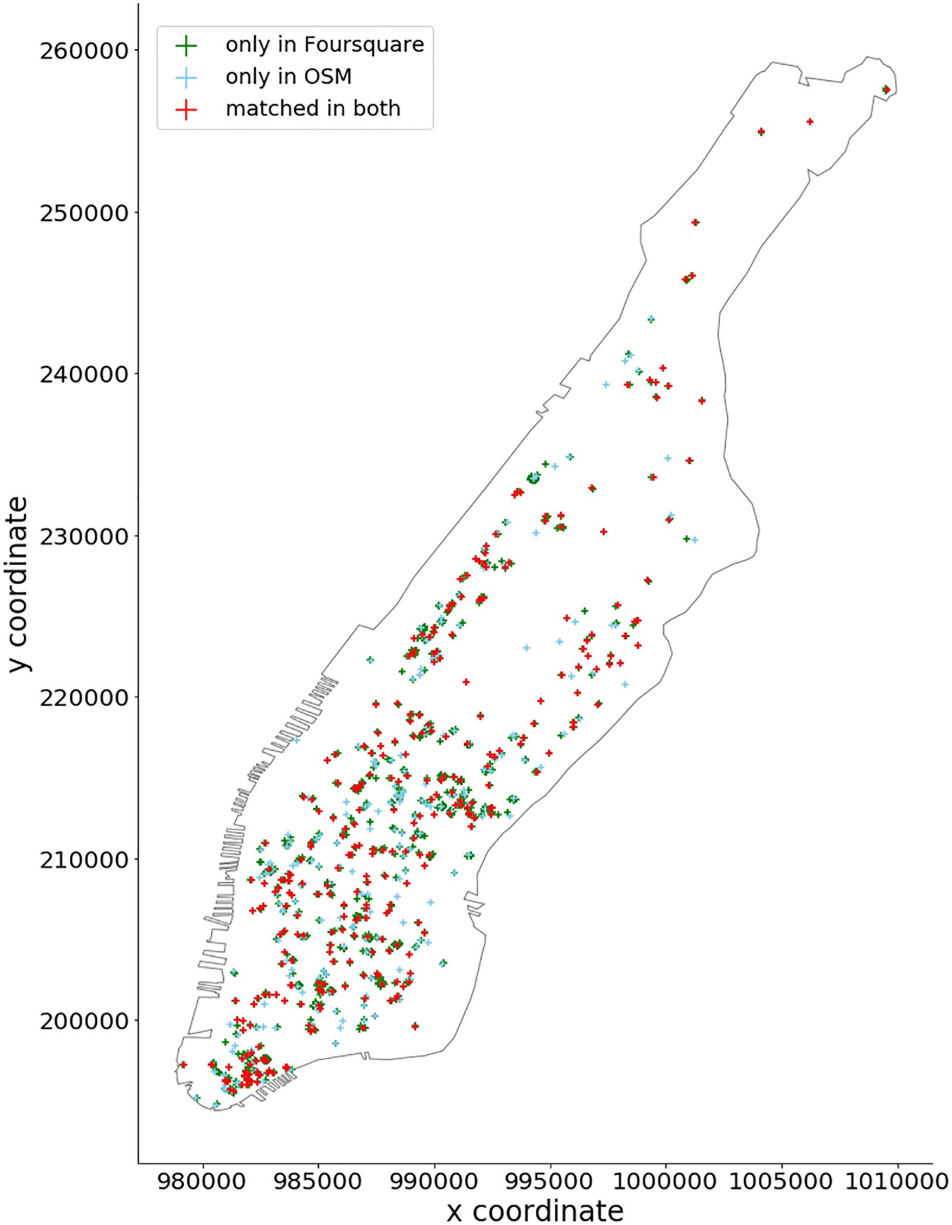
Result of POI accuracy and coverage assessment.

**Fig 13:**
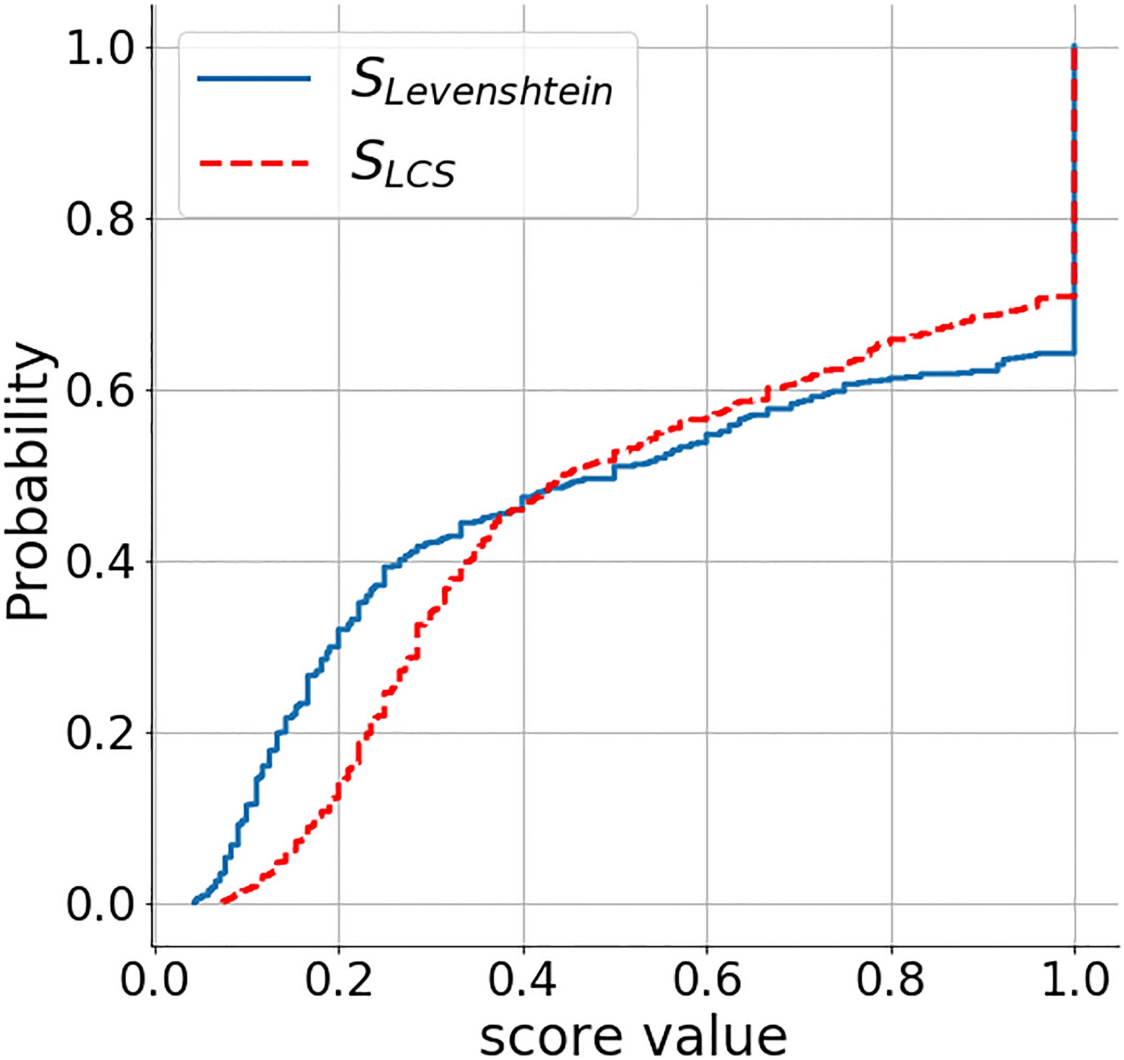
CDF of *S*_*LCS*_, *S*_*LD*_ (proximity threshold ≤ 50*m*).

**Fig 14:**
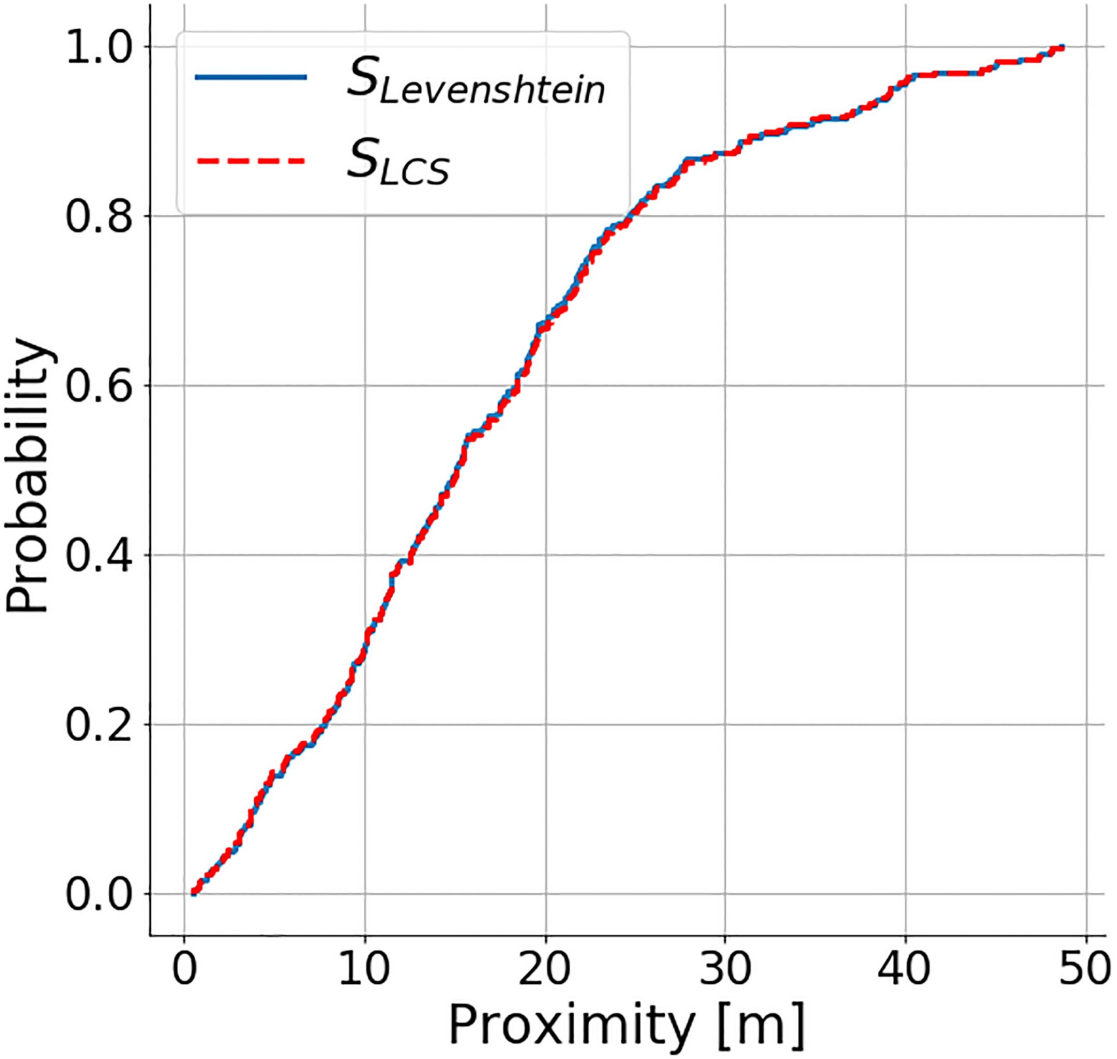
CDF of proximity (*S*_*LCS*_, *S*_*LD*_ ≥ 0.9, proximity threshold ≤ 50*m*).

The OSM dataset extract contains 529 coffee shops, while we were able to retrieve 851 locations from Foursquare. While the former strictly contains coffee shops, so does the latter also include other types of venues (e.g., sandwich and donut shops) due to the API characteristics. Using a strict label similarity score threshold of 0.9 and a 50*m* proximity threshold for location-matching, we were able to match 310 (LCS method) and 316 (Levenshtein method) OSM POIs to Foursquare data. Out of the 561 unmatched Foursquare POIs, we were able to match 210 (LCS) and 135 (Levenshtein) of them to other POIs in OSM. Overall 351 (LCS) or 426 (Levenshtein) Foursquare POIs and 219 (LCS) or 394 (Levenshtein) OSM POIs could not be matched to a respective equivalent in the other data source. The matched OSM locations are shown in the Fig 12. To see the distribution of the label similarity score *S*_*LCS*_, *S*_*LD*_ and location proximity, Fig 13 shows the CDF of the label matching score for pairs of names with distance threshold < 50*m*. We can see that about 35% of them have *S*_*LCS*_ = 1, which is much better than expected. It means that about 35% of the data can be matched exactly within 50*m*. Only 30% of the data has *S*_*LD*_ = 1. Fig 14 shows the CDF for label pairs with a proximity threshold < 50*m*. Close to 80% of all matching labels are within 30*m* of each other, and about 95% are within 40*m* for both methods. It tells us that the matched labels for both methods have a spatial accuracy of 30*m* − 40*m*.

### 5.2 Fitting scaling relationships

**Fig 15:**
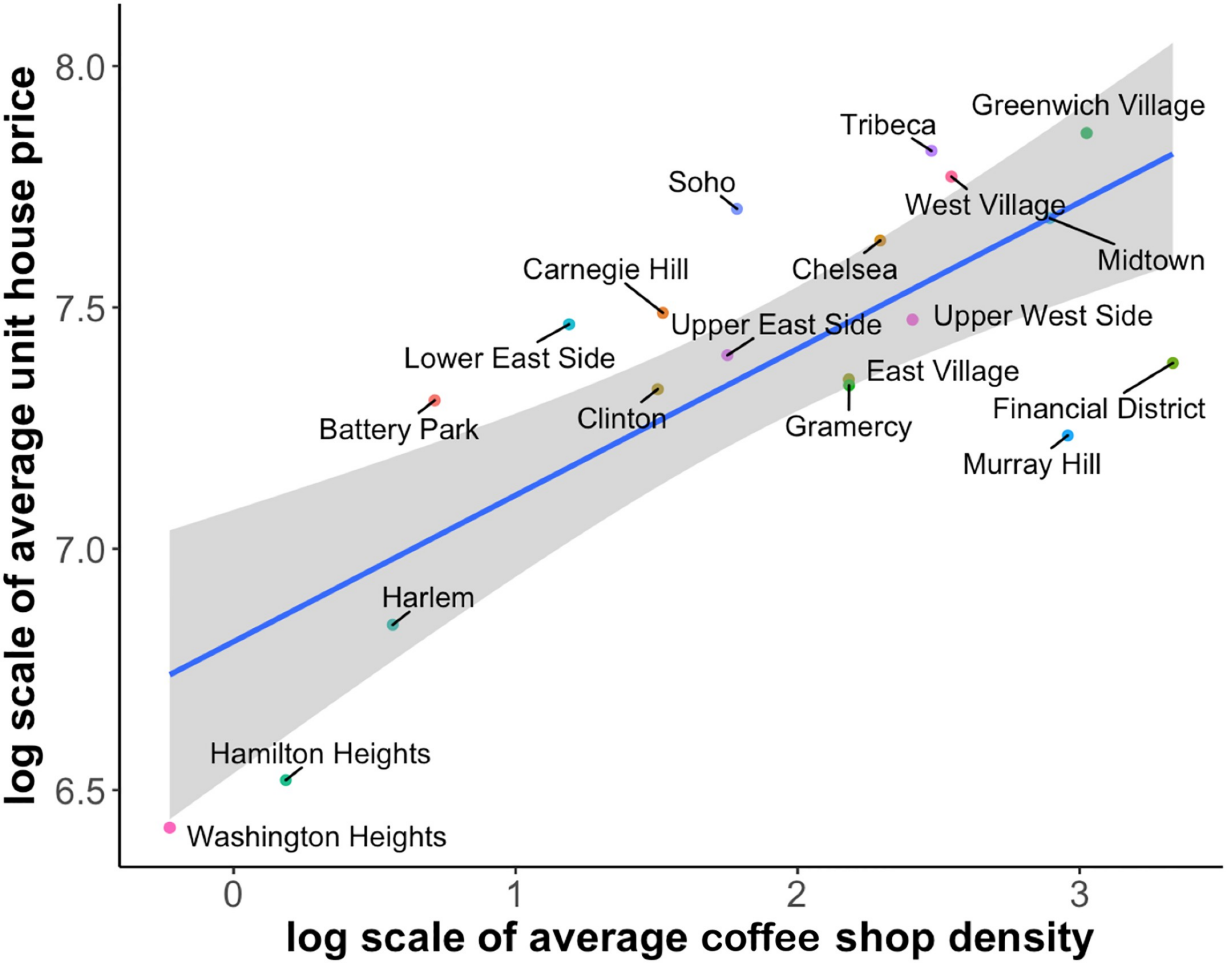
Coffee shop density means vs. home price means for the various neighborhoods.

**Fig 16:**
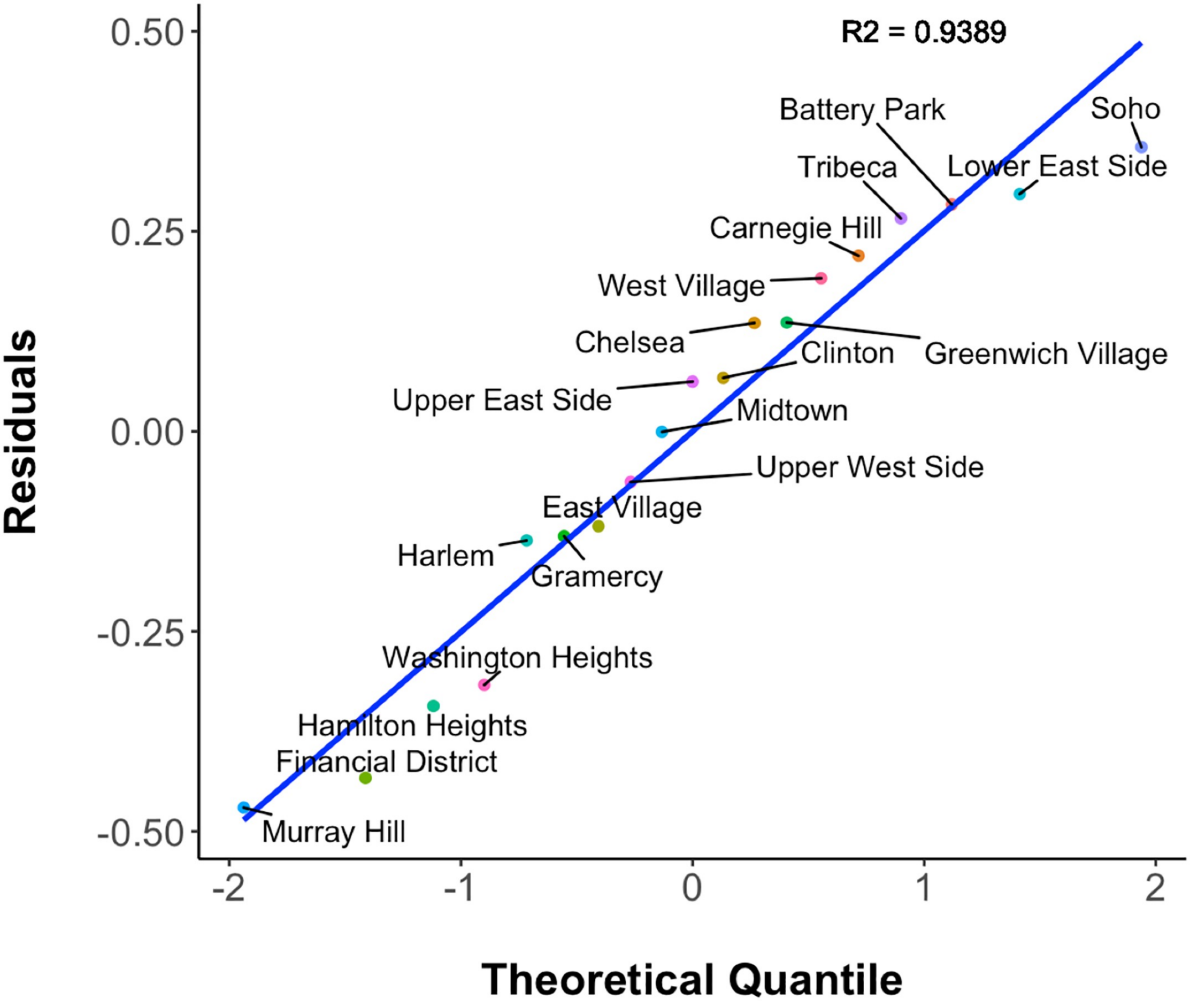
Q-Q plot of residuals of scaling relationship model.

For our first model case, we try to fit one scaling relationship model per season. Additionally, we use one baseline model for the mean value of coffee shop densities and home prices across seasons. The two variables are shown in Fig 15. The blue line is the fitted regression line. The grey area is the confidence interval of the predicted values. The coefficients and model fitting results of Table 1 suggest that across different seasons the scaling factor *β* is stable at around 0.30. All the models have very small p-values (less than 0.01), which indicates a good fit. *These results are a strong indication towards the existence of a scaling relationship between coffee shop densities and home prices*. Fig 16 shows the normal probability plot of residuals, which follows a normal distribution with a high goodness-of-fit (R-squared = 0.9389).

**Table 1:** Coefficients and p-values of basic scaling relationship model.

Models at different seasons	*β*	*log*(*Y*_0_)	p-value
Winter 2014	0.31	6.85	0.00058
Spring 2015	0.29	6.85	0.00101
Summer 2015	0.32	6.77	0.00053
Fall 2015	0.33	6.76	0.00057
Winter 2015	0.30	6.82	0.00077
Sprint 2016	0.30	6.80	0.00038
Summer 2016	0.30	6.79	0.00038
Fall 2016	0.31	6.77	0.00030
Mean	0.30	6.80	0.00047

Choosing *β* ≈ 0.3 is a reasonable value, since this scaling relationship model is based on the coffee shop density vs. population and home price vs. population models (cf. Eq 5). The addition of one coffee shop would imply the addition of a sizeable population. Thus, our *β* is considerably smaller than the values identified in [6], which are directly related to population. Several weaknesses of our basic model are also evident. About half of the points are outside of the confidence interval, and the changing temporal pattern inside one neighborhood in Fig 10 is in stark contrast to the stable spatial pattern of one season in Fig 11. We will address this issue in the models when considering adjustments.

### 5.3 Model results with adjustments

Section 4 presented a series of models, which added spatiotemporal variables or spatiotemporal methods to the basic model. We use the labels M1, M2, etc. to distinguish the various models (cf. Table 2). The coefficients of parameters, p-values, AIC, and BIC of each model are shown in Table 3.

**Table 2:** Abbreviation of different models.

abbreviation	modeling components
M1	Basic power law scaling relationship model
M2	Model with temporal trend
M3	Model with temporal lag
M4	Model with temporal and spatial trend
M5	Model with temporal and spatial trend, and spatial lag
M6	Model with temporal and spatial trend, and spatial error

**Table 3:** Estimated model parameters with adjustments.

Models with adjustments	Coefficients (p-value)	AIC/BIC	Models with adjustments	Coefficients (p-value)	AIC/BIC
M1	$\begin{array}{cc} y_{0} & =6.8067(\approx 0)\\ \beta & =0.3032(\approx 0) \end{array}$	19.8/28.7	M5	$\begin{array}{cc} \beta & =0.1895(\approx 0)\\ \alpha_{1} & =1.1639e-04(\approx 0)\\ \alpha_{2} & =-1.0865e-05(\approx 0)\\ \omega_{1} & =6.703(\approx 0)\\ \omega_{2} & =6.652(\approx 0)\\ \omega_{3} & =6.602(\approx 0)\\ \omega_{4} & =6.627(\approx 0)\\ \theta_{1} & =-6.9627e-05(\approx 0)\\ \theta_{2} & =2.2269e-05(0.018)\\ \theta_{3} & =9.0755e-08(0.004)\\ \theta_{4} & =1.5726e-08(0.001)\\ \theta_{5} & =-7.7248e-08(\approx 0)\\ \rho & =-0.0055(0.0163) \end{array}$	-65.3/-30.8
M2	$\begin{array}{cc} \beta & =0.1824(\approx 0)\\ \alpha_{1} & =1.326e-04(\approx 0)\\ \alpha_{2} & =-1.234e-05(\approx 0)\\ \omega_{1} & =6.622(\approx 0)\\ \omega_{2} & =6.548(\approx 0)\\ \omega_{3} & =6.495(\approx 0)\\ \omega_{4} & =6.520(\approx 0) \end{array}$	-40.9/-16.8			
M3	$\begin{array}{cc} y_{0} & =6.8088(\approx 0)\\ \beta & =0.3057(\approx 0)\\ \eta_{1} & =-0.0810(0.834)\\ \eta_{2} & =-0.1623(0.640)\\ \eta_{3} & =-0.0401(0.888) \end{array}$	18.0/33.3			
M4	$\begin{array}{cc} \beta & =0.2042(\approx 0)\\ \alpha_{1} & =1.136e-04(\approx 0)\\ \alpha_{2} & =-1.071e-05(\approx 0)\\ \omega_{1} & =6.551(\approx 0)\\ \omega_{2} & =6.490(\approx 0)\\ \omega_{3} & =6.442(\approx 0)\\ \omega_{4} & =6.466(\approx 0)\\ \theta_{1} & =-0.1488(\approx 0)\\ \theta_{2} & =-0.1808(0.002)\\ \theta_{3} & =1.27e-07(\approx 0)\\ \theta_{4} & =2.004e-08(\approx 0)\\ \theta_{5} & =-1.015e-07(\approx 0) \end{array}$	-61.5/-35.5	M6	$\begin{array}{cc} \beta & =0.1508(\approx 0)\\ \alpha_{1} & =1.839e-04(\approx 0)\\ \alpha_{2} & =-1.671e-05(\approx 0)\\ \omega_{1} & =6.484(\approx 0)\\ \omega_{2} & =6.334(\approx 0)\\ \omega_{3} & =6.263(\approx 0)\\ \omega_{4} & =6.293(\approx 0)\\ \theta_{1} & =-6.4873e-05(0.001)\\ \theta_{2} & =2.2995e-05(\approx 0)\\ \theta_{3} & =8.7868e-08(\approx 0)\\ \theta_{4} & =1.4554e-08(\approx 0)\\ \theta_{5} & =-7.0493e-08(\approx 0)\\ \lambda & =0.17997(\approx 0) \end{array}$	-77.3/-33.6

As mentioned in Section 4.2.1, **M1** is the basic model considering only coffee shop density as independent variable. *β* is estimated to be 0.3032, which is almost the same as in the case of the simple model using mean values. We will use this model as a baseline in our comparison to assess the various adjustments.

**Fig 17:**
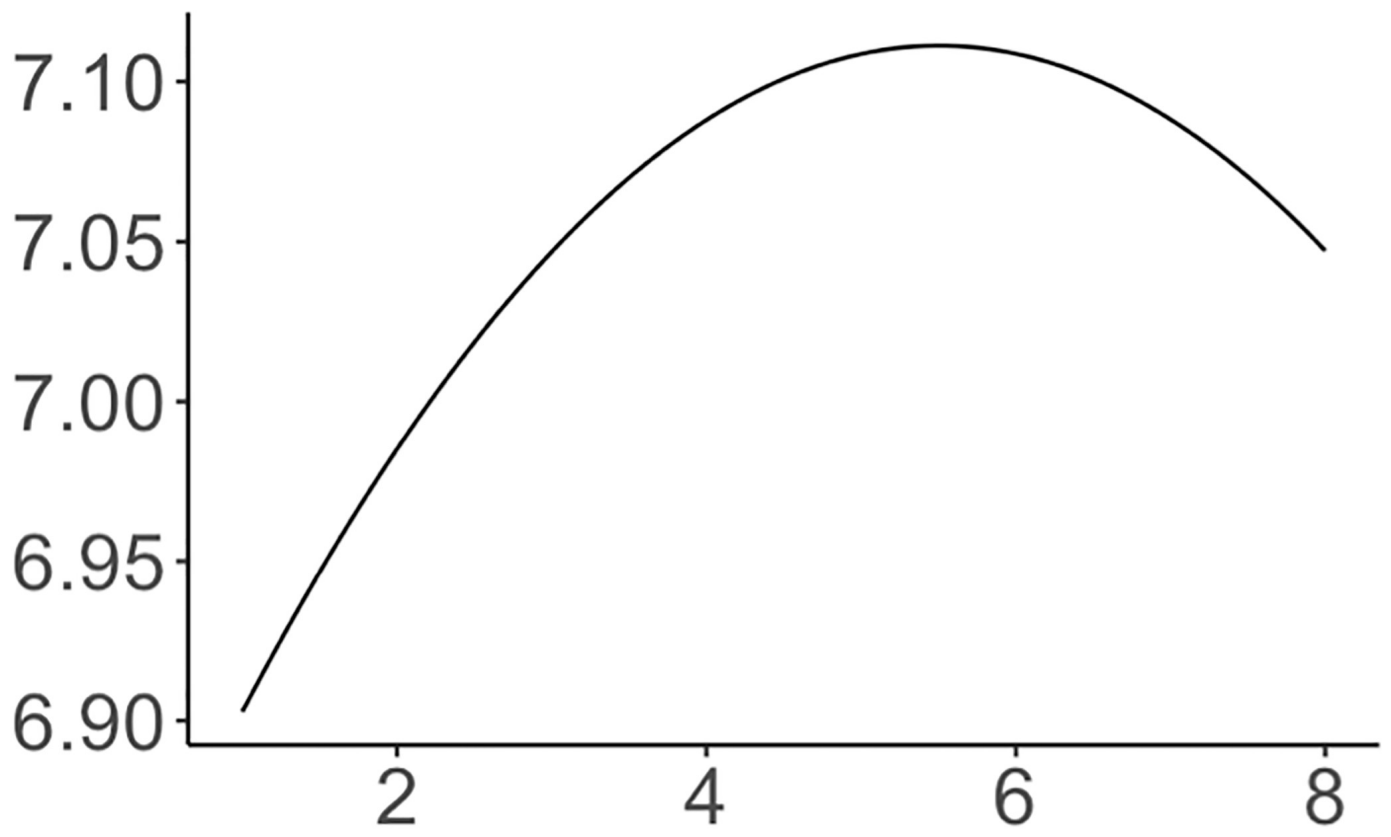
Estimated function of global temporal trend.

**Fig 18:**
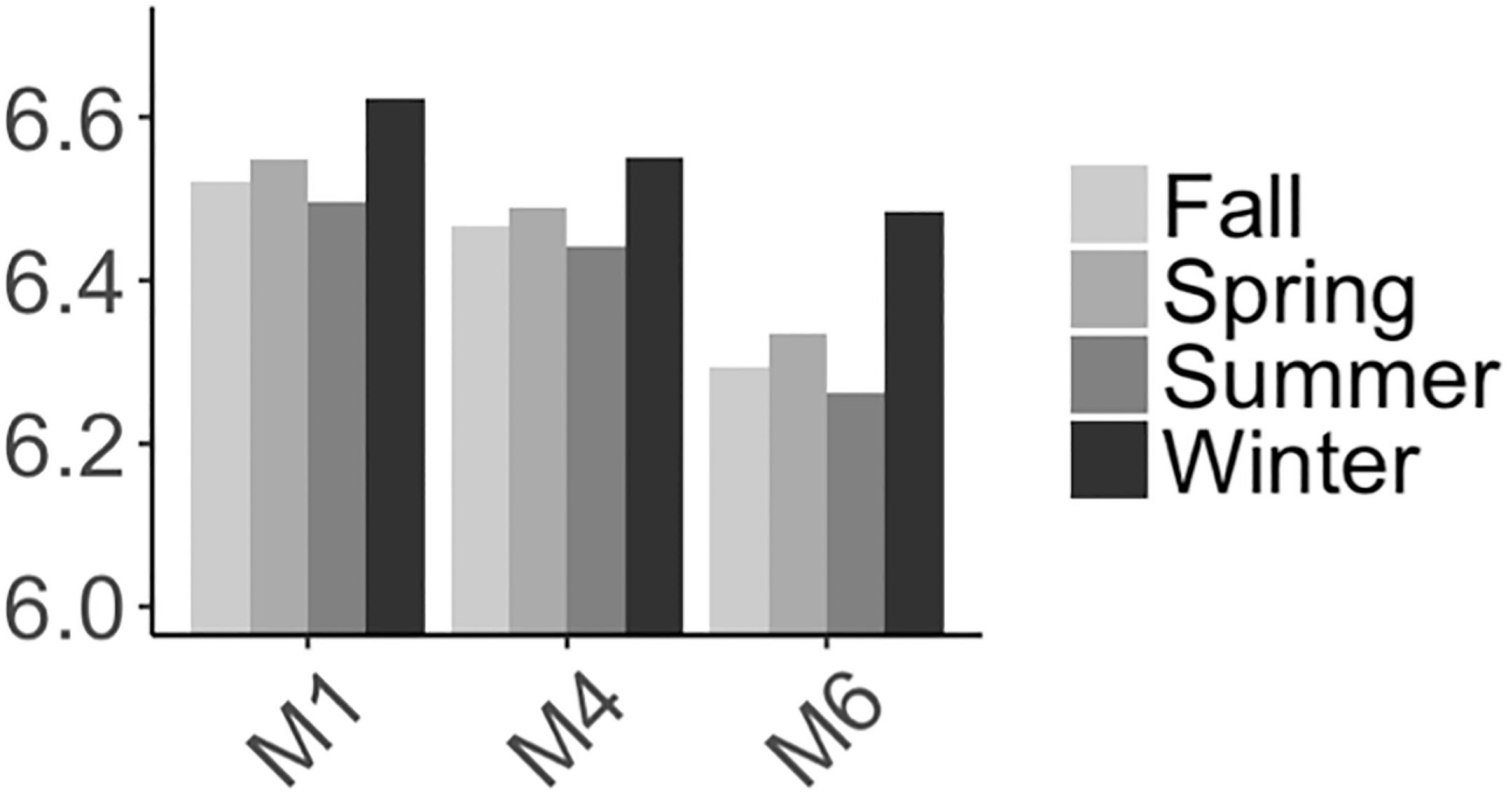
Estimated coefficients of seasonal trend of Manhattan.

*Temporal trend modeling* (**M2**) has coefficients that are all significant and with p-values close to 0. Here, *α*_1_ and *α*_2_ represent global trends. To understand what that means, we can assign an arbitrary coffee shop density value (2) and seasonal dummies (winter) to this model. The estimated global trend is shown in Fig 17. During those two years, real estate markets reached a peak, exhibiting a growth pattern and only shrinking somewhat towards the end of the period. The coefficient of coffee shop density, *β*, drops from 0.3032 in the basic model to now 0.1824. It shows that probably this global market change is correlated with the coffee shop density’s overall change during the observation period. The model also captures seasonality. With *w*_1_ representing winter, this means that home prices in winter would have a higher value than during other seasons (cf. Fig 18). For our different models, this seasonal pattern is the same across all models. However, it is in contrast to some other reports, e.g., [56], which claim that prices are lowest during winter. Limited by our available sparse data (only two years) and methods, we cannot conclusively explain this difference, only that it is statistically significant. Moreover, this model has an AIC value of -40.9 and a BIC value of -16.8, which is a significant decrease from M1’s AIC and BIC values of 19.8 and 28.7 respectively. As such, we are quite confident that global trends and seasonality impact our model.

Temporal lag modeling (**M3**) did not perform well, since all of the lag parameters have significant p-values, even though AIC slightly dropped to 18.0 (from 19.8 in M1), BIC is 33.3 (increase from 28.7 in M1). This also shows that **M3** does not improve over **M1**. Changes to coffee shop density have no effect beyond a single season (three months period).

**Fig 19:**
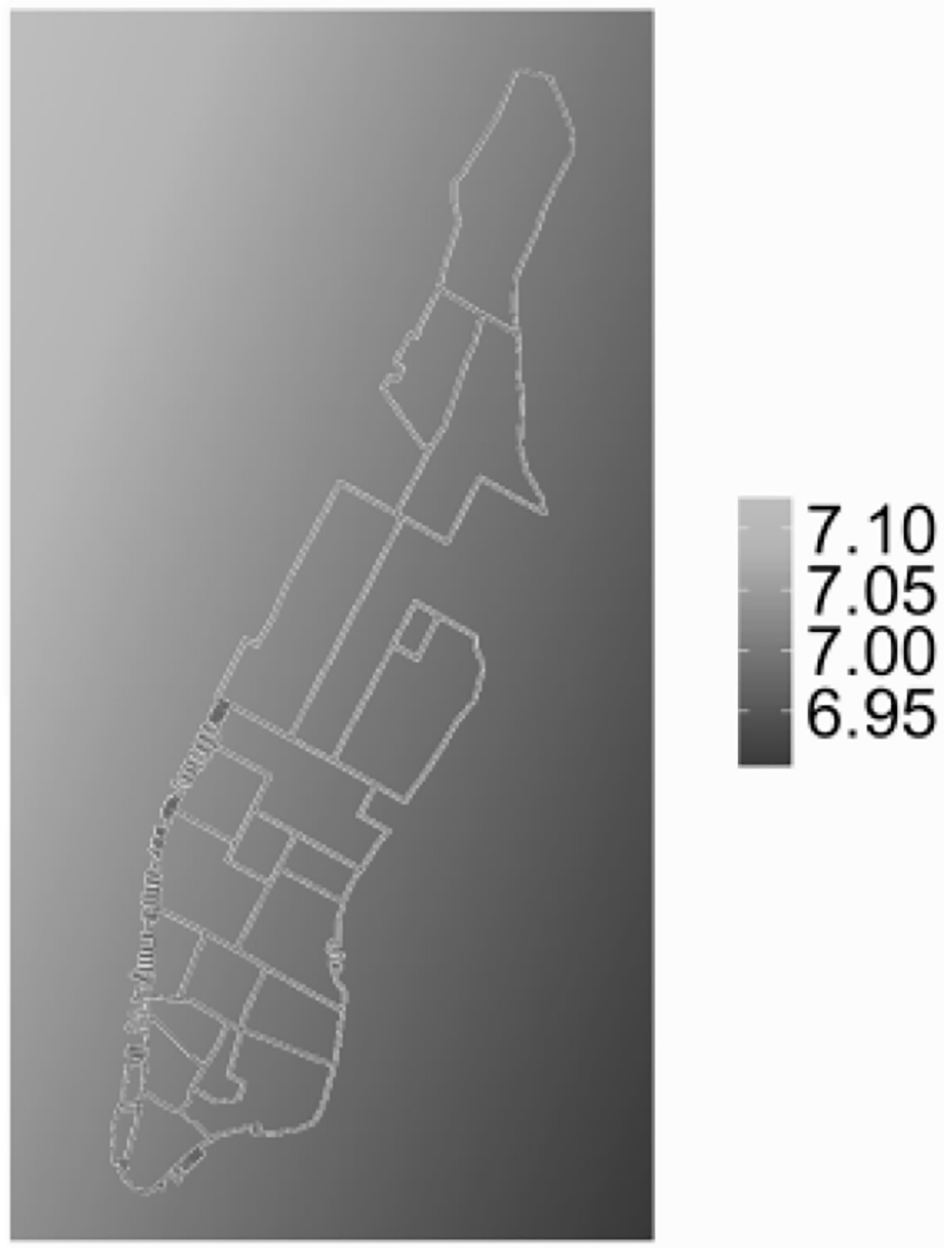
Estimated function of spatial trend of Manhattan.

Another interesting aspect is a potential spatial trend, i.e., does Manhattan’s home price follow a two-dimensional distribution? **M4** looks at this question. As we can see in Table 3, all *θ*s are significant. Assigning arbitrary values to all the other parameters, we can generate a trend surface as shown in Fig 19. It shows a slope pattern with higher values in the Northwest area and lower values towards Southeast. The result does not seem to be intuitive at first. Assuming it is not a fundamental issue with the modeling methods themselves, then a possible explanation is that only three neighborhoods in this study are located to the north of Central Park. Thus, the model is geared towards the trend in the southern area. Neighborhoods around Central Park typically also have high home prices. The overall power of this model improves considerably when compared to the next best one, M2, with AIC/BIC dropping to -61.5/-35.5 respectively. The coffee shop coefficient *β* = 0.2042 is only slightly higher than the 0.1824 for the case of M2. Still, this model seems to be quite a good fit.

**M5** and **M6** consider a *spatial autocorrelation effect*. Spatial autoregression is widely used in home price analysis. However, it might implicitly estimate latent factors, which are also estimated by coffee shop densities and spatial trends. The modeling results strongly support this effect. To see whether M5 and M6 capture similar spatial effects, we observe that both models have lower AIC/BIC, -65.3/-30.8 for M5, and -77.3/-33.6 for M6. This means that they are both statistically good models. The variables in both models have very low p-values, which indicates that they explain their effects very well. The *θ* coefficient values capturing spatial trends in both M5 and M6 are now considerably smaller (10^4^×) when compared to M4. This means that spatial autoregression has taken over the interpolation power from spatial trends. The coffee shop density coefficient *β* is also smaller. However, **M5** and **M6** do have some differences. The autoregression coefficient, *ρ*, of M5’s dependent variable is negative. This indicates a negative autocorrelation between neighbors. However, the error term’s coefficient, λ, is positive, which suggests a positive effect between neighbors that is unexplained by coffee shop density or other trends. The AIC/BIC value of M6 is -77.3/-33.6. Both are lower and indicate an improvement over M5. It is hard to say which model, M5 or M6, is right or wrong, since both autoregression models work on latent factors. The model itself did not explicitly give us information about the factors they estimate. Of course, there are other advanced modeling techniques that could be applied in future research and which might have more explanatory power.

### 5.4 Best model?

After comparing the p-values of coefficients, AIC and BIC, we can identify the two “best model” candidates. **M4** is the best model without an autoregressive process given its low BIC score. **M6** uses spatial autoregression and is the best in terms of overall AIC. **M6** has a worse BIC score, since it is penalized for its complexity by using autoregression terms.

**Fig 20:**
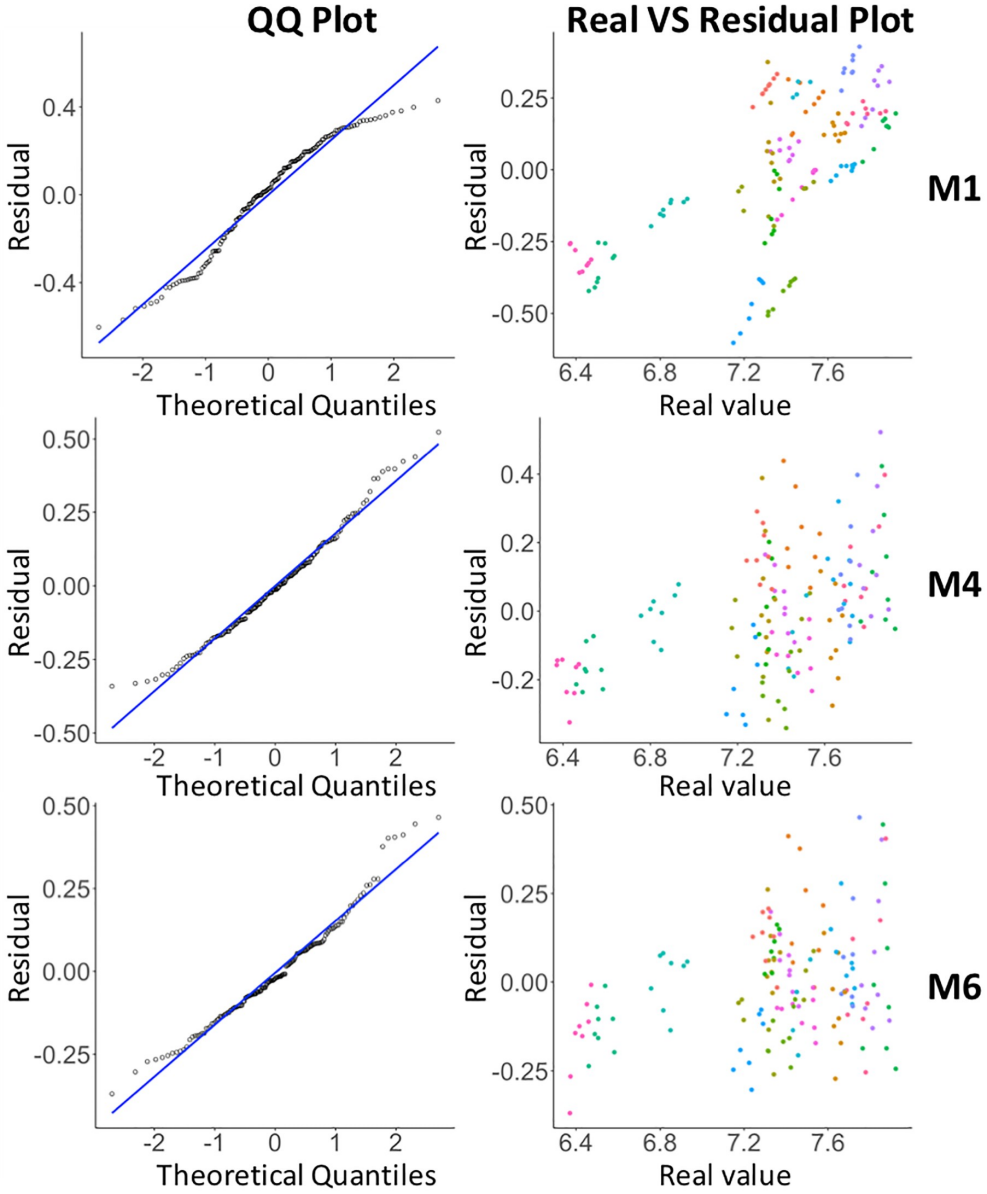
Residuals of fitting models: Comparison using Q-Q plots (left) and real vs. residual (right) for different models.

To visually compare the models in terms of prediction accuracy, we can use a Q–Q (quantile-quantile) plot, which is a probability plot that compares two probability distributions by plotting their quantiles against each other. Fig 20 plots the residuals of **M1**, **M4**, and **M6** against an expected normal distribution. M1 shows some deviation for both tails and also in the middle. The residuals for M6 are worse for the right tail portion. M4 seems to be the best of the three models. Its residuals fit to a normal distribution very well. However, M6’s total range of residuals (0.8341) is smallest, followed by M4 (0.8641) and M1 (1.032). Fig 20 shows for each neighborhood (points with the same color) that residuals fluctuate more for M6 than for M4 and M1. This is also the reason behind M6’s lowest AIC value. This pattern means a better fit to the normal distribution inside a neighborhood.

In general, based on our modeling approach, both M4 and M6 would be good candidates for modeling coffee shop densities in relation to home prices. With M6, even though this model has a better AIC score, there is a concern that the autoregressive process implicitly estimates the coffee shop density. This complexity is penalized as indicated by a larger BIC score than M4. The coffee shop density coefficient *β* = 0.1508, which is almost half of the *β* of the baseline model. For M4, *β* = 0.2043. Since it more convincingly considers coffee shop density, we consider *M4 to be the best overall model*.

### 5.5 Inverse coffee shop effects

The following power law based scaling relationship examines an intriguing, albeit intuitive observation about cities derived from our data. Using a simple model for mean home prices $P_{t}={\text{exp}}^{6.8}\times C_{t}^{0.3}$, a growth function can be obtained by calculating the derivative of the scaling relationship (cf. Eq 12). This growth function can help us in understanding the coffee shops’ contribution to local communities, i.e., how does adding one coffee shop affect home prices?

$$\begin{array}{c} \frac{dP_{t}}{dC_{t}}=\text{exp}\left(5.596\right)\times C_{t}^{-0.7} \end{array}$$ (12)

**Fig 21:**
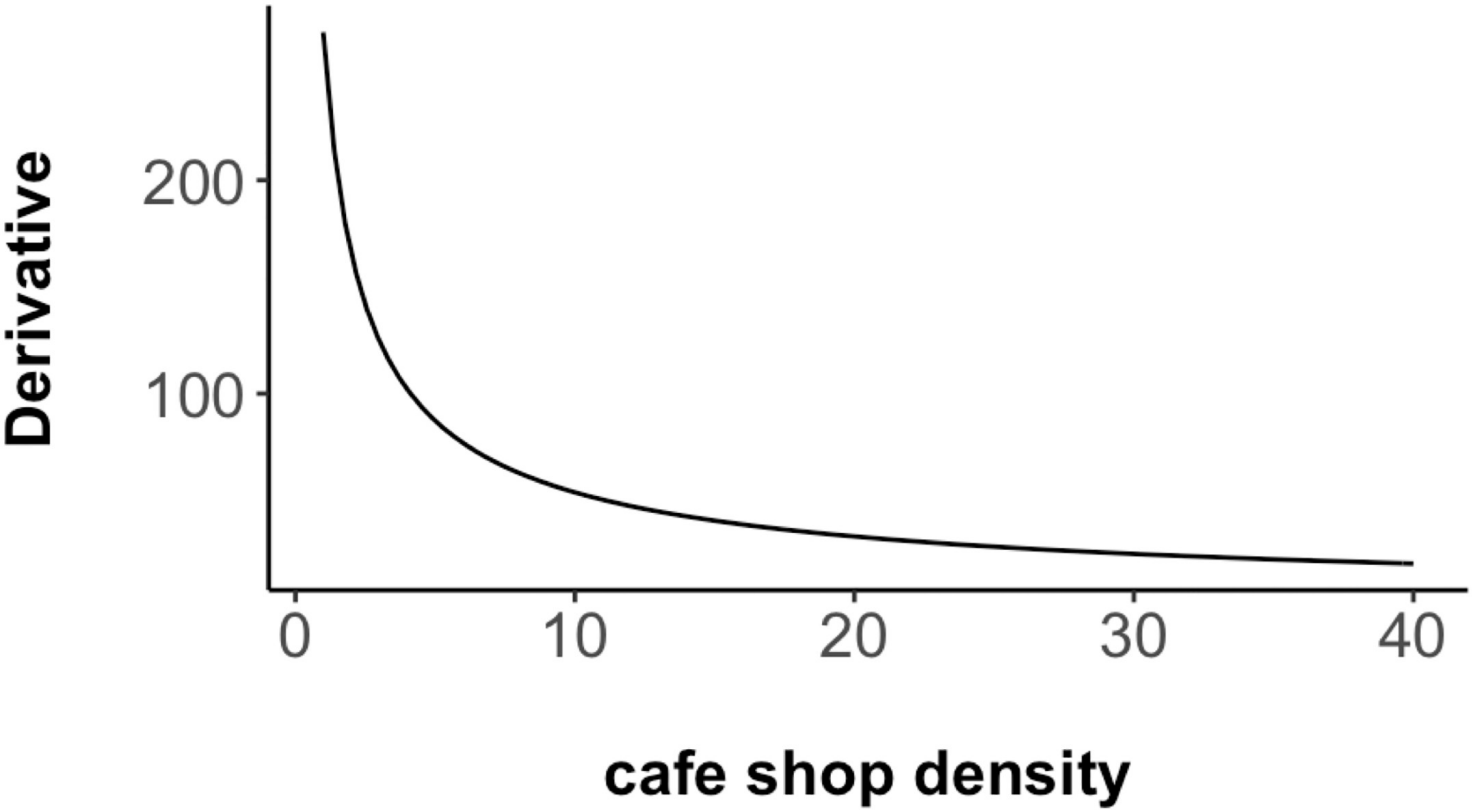
Derivative function of home price and coffee shop change.

Eq 12’s generic structure captures a fundamental feature of coffee shops and how they are related to neighborhood changes. The function shown in Fig 21 indicates that a change in coffee shop density has an inverse sublinear effect $C_{t}^{-0.7}$ on home prices. In underdeveloped neighborhoods with small coffee shop numbers (zero is not included in this discussion), home prices increase more rapidly when coffee shops are added. This effect corresponds to the left tail of the curve shown in Fig 21. In a highly developed neighborhood with many coffee shops, the impact of a new coffee shop is rather small. This also explains that the different *neighborhoods have different temporal correlations between coffee shop densities and home prices*. The lower left corner of Fig 10 captures neighborhoods in which home prices grow faster with the addition of coffee shops. The upper right corner in the same figure shows neighborhoods that have already a high concentration and the growth dynamic backed by cafe shops is less evident. This growth function now provides an explanation for this phenomenon. It also confirms the intuition that the first, for example, Starbucks coffee shop has the biggest impact on neighborhood development (Harlem vs. the Upper East Side). As such, our work supports that argument that one could really use coffee shop densities as an indicator for gentrification as suggested in [36].

## 6 Conclusions

The study of urban change has always been of critical importance and has only become widely feasible in recent years due to the availability of open data and user-generated content. However, the quality of such data for this particular use case still needs to be systematically examined as addressed in this work. By examining the accuracy and coverage of OSM POI data we found that it compares favorably to other more authoritative data sources. Compared to Foursquare, 60% of the OSM POIS could be matched with high accuracy. A more important aspect we looked at is the trend worthiness of the data, i.e., even if it is not as accurate, does it still capture temporal trends such as the relative increase of coffee shops over time? Using statistical models that exploit the power law relationship of various factors in relation to population, we were able to relate coffee shop POI data to urban housing prices. The models that we derived allow us to show that even though OSM POI data (coffee shops in our case) might be incomplete, i.e., not all coffee shops are recorded in a timely matter, such data can still be used in urban analytics research. It is also interesting to observe that the estimated growth function decodes a generic process of urban change and shows that coffee shop data can be an important geo-social indicator.

We can give the following directions for future research. We can apply our models to evaluate further user-generated content datasets, such as restaurants, supermarkets, parking garages, and bike sharing systems. A goal could be to define a respective data matrix that shows which aspects are best suited to predict urban change. On the other hand, we want to extend this model to include all kinds of different urban phenomena and datasets including, but not limited to crime, street vitality, and local business climate. Further, we want to improve our spatiotemporal modelling techniques. Various approaches exist that rely on more advanced statistical models, such as spatial-temporal autoregression [57], and spatial filtering [52]. Overall, although we focused on a very specific model in this work, we argue that the proposed approach (modeling plus user generated content) has considerable potential for social science research.

## Supporting information

S1 Appendix Categories mapping and query details. (PDF)
