# Supplementary material for: Using OpenStreetMap point-of-interest data to model urban change—A feasibility study
Source: PLoS One. 2019 Feb 25;14(2):e0212606. doi: 10.1371/journal.pone.0212606 (PMC6388917; doi:10.1371/journal.pone.0212606)
Supplement: S1 Appendix — (PDF) [file pone.0212606.s001.pdf]

# Appendix

## Query details

To retrieve the respective POIs, we used the following query parameters for the respective services.

**OSM Changeset Query** - The raw OSM dataset is pre-processed using a range of tools (cf. https://github.com/MaZderMind/osm-history-renderer) and saved in a PostgreSQL database. We use SQL to query the database and to retrieve the changeset records. We retrieve all changeset records that contain the keywords "cafe", "cafeteria" or "coffee".

**Foursqure API Query** - the Venue API of Foursquare provides a "search" endpoint (https://developer.foursquare.com/docs/api/venues/search). The parameters we used are shown in Tables A and B. For Type II queries (retrieving Foursquare POIs around OSM POIs), we did not use category information so as to maximize the number of retrieved candidates.

**Table A.** Type I query parameters, Foursquare Venue API

| Parameter Name | Setting | Description |
|---|---|---|
| query | OSM POI name | approximate match |
| intent | browse | find venues around OSM POIs |
| Type II API call | OSM POI coords | buffer region |
| radius | 50$m$ | radius |
| limit | 50 POIs | number of results, up to 50 |

**Table B.** Type II query parameters, Foursquare Venue API

| Parameter Names | Setting | Description |
|---|---|---|
| categoryId | unique IDs shown in Table A | - |
| intent | browse | find venues in area (grid cell) |
| Type II API call | grid center | buffer region |
| radius | 50$m$ | radius |
| limit | 50 POIs | number of results, up to 50 |

## OSM and Foursquare POI Categories

The POI categories in Foursquare and OSM differ and as such we manually check and match them in our experiments, i.e., to retrieve the respective POIs from OSM and the Foursquare API. The mapping between OSM and Foursquare categories is shown in Table C.

**Table C. Categories mapping parameters**

| Source | Category | Unique ID |
|---|---|---|
| OSM | cafe | N/A |
| Foursquare | food/Cafeteria | 4bf58dd8d48988d128941735 |
| | food/Cafe | 4bf58dd8d48988d16d941735 |
| | food/donut | 4bf58dd8d48988d148941735 |
| | office/Coporate Cafeteria | 54f4ba06498e2cf5561da814 |
